# Impact of Non-Malignant Portal Vein Thrombosis in Recipients with Metabolic Dysfunction-Associated Steatotic Liver Disease Compared to Other Transplant Indications

**DOI:** 10.3390/jcm15051787

**Published:** 2026-02-27

**Authors:** Esli Medina-Morales, Yash Shah, Anastasia Xynogala, Mohamed Ismail, Ritik M. Goyal, Yazan Abboud, Hirsh D. Trivedi, Thomas D. Schiano, Keri E. Lunsford

**Affiliations:** 1 Department of Medicine, Rutgers New Jersey Medical School, Newark, NJ 07103, USAritik5678@gmail.com (R.M.G.);; 2 Division of Transplant and HPB Surgery, Department of Surgery, Rutgers New Jersey Medical School, Newark, NJ 07103, USA; ys880@njms.rutgers.edu (Y.S.); ax55@njms.rutgers.edu (A.X.); 3 Division of Gastroenterology and Hepatology, Cedars-Sinai Medical Center, Los Angeles, CA 90048, USA; hirsh.trivedi@austin.utexas.edu; 4 Mount Sinai Medical Center, New York, NY 10029, USA; 5 Center for Immunity and Inflammation, Rutgers Biomedical and Health Sciences, Newark, NJ 07107, USA

**Keywords:** liver transplantation, metabolic dysfunction-associated steatohepatitis, metabolic dysfunction-associated steatotic liver disease, non-alcoholic fatty liver disease, portal vein thrombosis, donation after circulatory death

## Abstract

**Background/Objectives**: Metabolic dysfunction-associated steatotic liver disease (MASLD) is associated with an increased risk of portal vein thrombosis (PVT), which may negatively affect post-liver transplant (LT) outcomes. We aimed to evaluate the impact of PVT on post-LT outcomes in MASLD versus non-MASLD recipients and assess outcomes in MASLD patients with PVT who received donation after circulatory death (DCD) grafts. **Methods**: Using the UNOS database, we analyzed adult LT recipients from 2002 to 2022. Kaplan–Meier and Cox regression models were used to assess one-year post-LT outcomes. **Results**: Among 46,933 LT recipients, 20% had MASLD (15% PVT prevalence) and 80% had non-MASLD etiologies (9% PVT prevalence). Overall, 3051 recipients (6.5%) received DCD grafts. PVT at the time of transplant was associated with significantly higher risks of all-cause mortality, graft failure, and death-censored graft failure (DCGF) in both MASLD and non-MASLD groups (*p* < 0.05), although no significant differences were observed between the two groups. In the DCD subgroup, MASLD recipients with PVT had a significantly higher risk of all-cause mortality compared to non-MASLD recipients without PVT (adjusted hazard ratio [aHR] 2.24, 95% CI 1.17–4.28, *p* = 0.01), but no differences were observed for graft failure or DCGF. **Conclusions**: PVT at the time of transplant is associated with poorer survival in MASLD and non-MASLD recipients. No difference was found between the two groups. In candidates receiving DCD grafts, the presence of PVT at time of transplant was associated with a marked increase in mortality risk, although this finding requires further validation in larger cohorts.

## 1. Introduction

The global prevalence of metabolic dysfunction-associated steatotic liver disease (MASLD) has surged, affecting nearly one-third of the adult population and emerging as the fastest-growing indication for liver transplantation (LT) [1,2]. MASLD is now the second leading LT indication in the United States, mirroring European trends [2,3]. These trends underscore the critical importance of understanding MASLD’s impact on LT indications and outcomes.

MASLD is increasingly being recognized as a prothrombotic condition. The persistent inflammation associated with MASLD triggers heightened activation of the coagulation cascade, characterized by reduced protein C and fibrinolysis, alongside elevated factor VIII [4]. This inflammatory state, with the concurrent presence of diabetes and obesity, significantly increases the risk of thrombotic events in MASLD patients, including portal vein thrombosis (PVT) [5,6,7].

PVT at the time of LT has been linked to increased early post-transplant morbidity and mortality, with a reported prevalence of 2% to 26 [8,9]. In MASLD recipients specifically, a recent study indicated that those with PVT at LT had a 31% higher risk of overall mortality and a 37% higher risk of graft failure compared to those without PVT [10]. Currently, the prevalence of PVT in MASLD recipients approaches 12% [11], which is double that of recipients without MASLD. As waitlist times continue to increase, it is crucial to investigate whether the presence of PVT in MASLD recipients is associated with worse post-transplant outcomes compared to non-MASLD recipients who also have PVT. The growing use of livers from donation after circulatory death (DCD) donors, prompted by ongoing organ shortages, adds another layer of complexity [12,13]. The combination of MASLD and PVT in recipients of DCD liver grafts raises significant concerns regarding surgical challenges, graft viability, and recipient survival.

This study aimed to (1) evaluate the impact of PVT on post-LT all-cause mortality, graft failure, and death-censored graft failure (DCGF) in MASLD versus non-MASLD recipients; and (2) assess outcomes of MASLD recipients and PVT who underwent LT with DCD grafts. We hypothesized that the presence of PVT would worsen post-transplant outcomes in MASLD recipients compared with non-MASLD recipients with PVT, and this effect would be exacerbated in recipients of DCD liver allografts.

## 2. Materials and Methods

### 2.1. Data Source and Cohort Definition

A retrospective cohort study was conducted utilizing OPTN database, which was overseen by the U.S. Department of Health and Human Services. The OPTN contractor, the UNOS, is responsible for its operations. Institutional Review Boards at Rutgers New Jersey Medical School (NJ, USA), Cedars-Sinai Medical Center (CA, USA), and Mount Sinai Hospital (NY, USA) deemed the UNOS database de-identified and publicly available, thus exempt from approval. The data reported here have been supplied by UNOS as the contractor for the OPTN. The interpretation and reporting of these data are the responsibility of the author(s) and in no way should be seen as an official policy of or interpretation by the OPTN or the U.S. Government.

Based on OPTN data as of February 2022, our study focused on LT recipients aged 18 years or older who underwent transplantation from February 2002 to February 2022. Exclusion criteria included: (1) multi-organ transplant—including simultaneous kidney–liver; (2) split-liver allografts; (3) prior LT; (4) status 1; (5) living donor LT; (6) prior transjugular intrahepatic portosystemic shunt (TIPS); (7) history of malignancy, including cholangiocarcinoma, hepatoblastoma, or hepatocellular carcinoma; (8) transfer or transplant at another center after listing, to prevent double-counting of patients; and (9) unknown PVT status at the time of transplant.

Our study cohort comprised patients with the primary diagnosis of MASLD and other chronic liver conditions (non-MASLD cohort), including alcoholic liver disease (ALD), autoimmune liver disease (AILDs), hepatitis B (HBV), hepatitis C (HCV), and others. Secondary diagnoses were disregarded except for cryptogenic cirrhosis (diagnosis codes 4208 and 4213), which was included in the MASLD cohort if the secondary diagnosis corresponded to MASLD, following the methodology reported in other studies [3,14,15]. Codification for diagnosis can be found on Table S1.

### 2.2. Study Variables and Definitions

Our primary variable of interest was PVT at the time of transplant (PVT Tx), as documented by the center on the Transplant Recipient Report (TRR). We also defined the previous history of PVT (PVT Hx) in patients documented by the center to have PVT at the time of listing or registration (as reported on the Transplant Candidate Report [TCR]). Based on this data, the patient cohort was stratified based on PVT Tx and/or PVT Hx, following the approach outlined in previous studies [10]. Baseline covariates included recipient characteristics (age, sex, race/ethnicity, blood type), manifestations of portal hypertension (spontaneous bacterial peritonitis, encephalopathy, ascites), comorbidities (prior abdominal surgery, dialysis), severity of liver disease indicated by the Model for End-Stage Liver Disease (MELD) score at the time of transplant (most recent listing MELD), laboratory values (albumin, bilirubin, INR, creatinine, sodium), and risk factors for MASLD (body mass index [BMI] ≥ 30, diabetes). Donor covariates included donor characteristics (age, sex, race/ethnicity, BMI, BMI ≥ 30), presence of diabetes, cold ischemia time (CIT), liver donor risk index (DRI)—alone and categorized into tertiles, donor graft type (donation after brain death [DBD] or DCD), and donor–recipient match per body surface area (BSA), as previously described. Listed and transplant patients were subclassified by era in 5-year interval periods: 2002–2006, 2007–2011, 2012–2016, and 2017–2022. The UNOS regions were grouped into four geographic areas: Northeast (1, 2, and 9), Southeast (3, 4, and 11), Midwest (7, 8, and 10), and West (5 and 6).

Variables were considered as missing values under the following conditions: recipient characteristics (BMI < 15 or > 55 kg/m^2^), laboratory values (albumin < 0.5 or >6 g/dL, total bilirubin < 0.1 or >50 mg/dL, creatinine < 0.1 or >15 mg/dL), and donor variables (age < 10 or >75 years old, CIT < 1 or >24 h).

### 2.3. Outcome Assessment

The primary outcome was all-cause mortality. Secondary outcomes included graft failure and DCGF following LT. The follow-up period for all-cause mortality began on the date of LT and extended to the date of death. For graft failure, the follow-up period commenced on the date of LT and continued until either irreversible graft failure or patient death, whichever event came first. For DCGF, the follow-up period commenced on the date of LT and continued until irreversible graft failure.

### 2.4. Statistical Analysis

Baseline recipient and donor characteristics were categorized by the presence or absence of PVT at the time of transplant and by etiology of liver disease: MASLD and non-MASLD. Continuous variables were presented as means ± standard deviation (SD) (if normally distributed) or as median and interquartile range (IQR) and analyzed using the Student *t*-test or the Kruskal–Wallis test, as appropriate. Categorial variables were presented as frequencies and percentages and analyzed using the chi-square test.

Kaplan–Meier estimates were conducted to determine the effect of PVT at the time of transplant on post-LT outcomes within one year after transplantation. Overall patient survival, graft failure, and DCGF rates at 3, 6, 9, and 12 months post-transplant were compared using the log-rank test. Post-LT survival outcomes across 5-year interval periods were compared using the Peto-Peto-Prentice test [16]. Hazard ratios (HRs) and 95% confidence intervals (CI) were calculated using the Effron approximation for handling ties [14,17]. Cox proportional hazards modeling was performed to study the effect of PVT on post-LT outcomes. The same approach was used in the group analysis, including only liver recipients from DCD donors. Multivariable adjustments were made for recipient and donor covariates, with proportional-hazards assumptions tested using Schoenfeld residuals. To ensure compliance with proportionality, the model was adjusted by employing the “strata ( )” function in STATA, adjusting the baseline hazard function across the different levels of the covariates diabetes and obesity. Model fit was evaluated using Cox–Snell residuals. We further stratified analyses by sex status within the MASLD cohort to evaluate potential effect modifications associated with post-LT outcomes. Variables with a *p*-value < 0.20 and those clinically relevant were included in the models through forward manual selection.

Additional subgroup analysis examined the effect of PVT on post-LT outcomes based on its presence at listing and/or transplant. Causes of death and graft failure were also assessed. Statistical significance was defined as *p* < 0.05, with analyses conducted using Stata version 18.0 (StataCorp, College Station, TX, USA).

## 3. Results

### 3.1. Study Cohort

**Figure 1:**
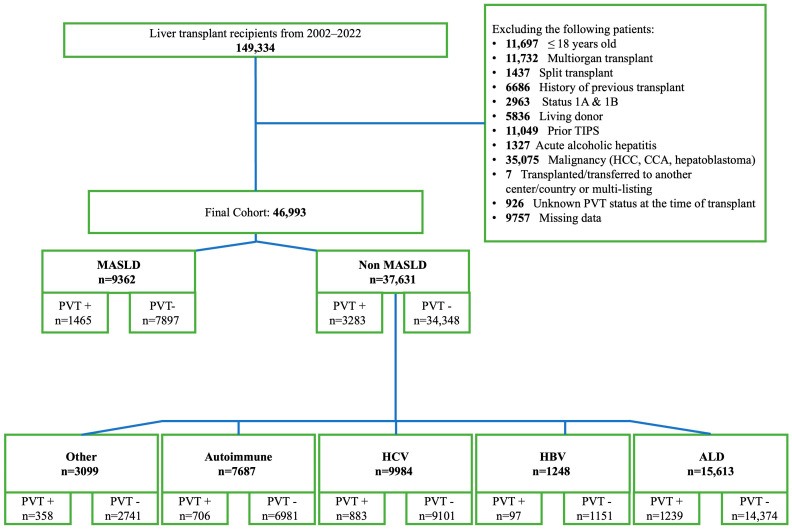
Study cohort flow chart. Note: Autoimmune disease included primary biliary cholangitis, primary sclerosing cholangitis and autoimmune hepatitis. Other included cryptogenic cirrhosis with body mass index lower than 30, Wilson disease, hemochromatosis and alpha-1 antitrypsin deficiency. Abbreviations: ALD, alcoholic liver disease; CCA, cholangiocarcinoma; HCC, hepatocellular carcinoma; HBV, hepatitis B; HCV, hepatitis C; MASLD, metabolic dysfunction-associated steatotic liver disease; PVT, portal vein thrombosis; TIPS, transhepatic intrajugular portosystemic shunt.

Of 149,334 adult LT recipients transplanted from February 2002 to December 2022 in the United States, a final cohort of 46,993 met all inclusion and exclusion criteria. Among these, 9362 (20%) had MASLD, while 37,361 (80%) had non-MASLD etiologies (Figure 1). Non-MASLD group etiologies included ALD (*n* = 15,613; 41.4%), HBV (*n* = 1248; 3.3%), HCV (*n* = 9984; 26.5%), AILDs (*n* = 7687; 20.4%), and other etiologies (*n* = 3099; 8.2%). PVT at the time of transplant was present in 15% of MASLD patients and 9% of non-MASLD patients.

Regardless of liver disease etiology, recipients with PVT at the time of transplant (PVT+) exhibited distinct clinical differences (Table 1). Compared to those without PVT (PVT−), MASLD PVT+ and non-MASLD PVT+ patients were older (*p* < 0.01, both), less likely to be female (*p* = 0.03, MASLD, *p* < 0.01, non-MASLD), and more likely to have diabetes (*p* < 0.01, both), a history of recent SBP (*p* < 0.01, both), or prior abdominal surgery (*p* < 0.01, both). Both MASLD and non-MASLD patients with PVT+ tended to be transplanted with lower biologic MELD scores (*p* < 0.01, both), as well as lower total bilirubin (*p* < 0.01, both), higher serum albumin (*p* = 0.04, MASLD and *p* < 0.01, non-MASLD), and higher sodium (*p* = 0.04, MASLD and *p* < 0.01, on-MASLD). Donors for MASLD and non-MASLD patients with PVT+ exhibited statistically higher BMI (*p* < 0.01, both). CIT was also greater for PVT+ recipients (*p* < 0.01, both), likely reflecting longer operative times. A higher proportion of non-MASLD PVT+ patients received DCD grafts compared to those without PVT (7.9% vs. 6.3%, *p* < 0.001).

### 3.2. Prevalence PVT in Study Cohort

The prevalence of PVT in MASLD recipients doubled over time, increasing from 8.8% in 2002–2006 to 17.2% in 2017–2022. This prevalence was notably higher in MASLD than in non-MASLD recipients, whether considered as a combined group or stratified by liver disease etiology (Figure S1A,B). Of note, the incidence of PVT among HCV+ recipients was also noted to rise across eras, with the highest incidence occurring following the introduction of Direct Acting Antivirals (DAA).

### 3.3. Effect of PVT on Post-Transplant Outcomes

#### 3.3.1. Kaplan–Meier Estimates of Post-Transplant Outcomes

**Figure 2:**
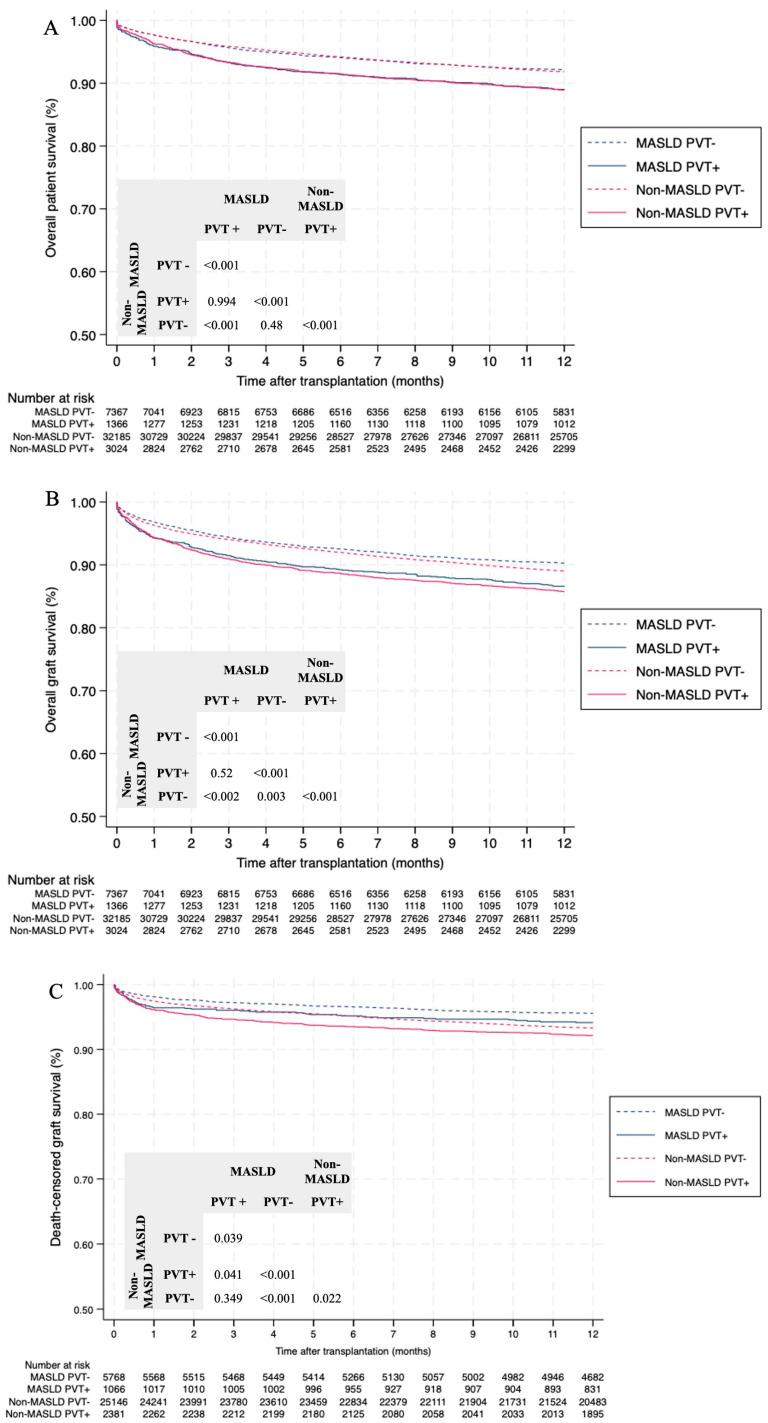
Kaplan–Meier curves of (**A**) overall patient survival, (**B**) graft survival and (**C**) death-censored graft survival after one-year post-LT in patients with MASLD and non-MASLD with and without PVT.

Presence of PVT at the time of transplantation (PVT+), regardless of liver disease etiology, was associated with poorer overall patient survival, graft survival, and death-censored graft survival (Figure 2A–C). MASLD and non-MASLD PVT+ recipients exhibited significantly lower patient survival, graft survival, and death-censored graft survival rates at 1, 3, 6, and 12 months post-transplant compared to those without PVT (*p* < 0.01) (Table 2). Notably, non-MASLD PVT+ recipients demonstrated lower death-censored graft survival rates compared to MASLD PVT+ recipients, (*p* = 0.041) (Figure 2C).

Next, the patient cohort was stratified into 5-year intervals to assess the impact of the transplant era on recipient outcomes. As expected, post-transplant outcomes in PVT+ recipients have improved over time (Figures S2–S4). MASLD PVT+ had worse patient survival and death-censored graft survival compared to MASLD PVT− across all periods, except for 2012–2016. Similarly, lower graft survival was observed in MASLD PVT+ in all periods, except for 2002–2006. During this era, relatively few cases of graft loss were reported in MASLD patients. Graft survival was lower in MASLD PVT+ compared with MASLD PVT− recipients (*p* = 0.125) in this era, but the difference was not significant. Non-MASLD PVT+ patients similarly exhibited significantly lower patient, graft, and death-censored graft survival rates across all time periods, except for death-censored graft survival during the 2002–2006 interval (Figures S2–S4).

Among non-MASLD PVT+, the lowest one-year post-LT survival outcomes were observed in HCV patients (patient survival: 86.9%, graft survival: 85.2%, and death-censored graft survival: 90%) and those in the “other” subgroup (patient survival: 85.2%, graft survival: 81.9%, and death-censored graft survival: 90%) (Figure S5).

#### 3.3.2. Subgroup Analysis Assessing the Effect of PVT at Listing and/or Time of Transplantation

We next evaluated the impact of PVT at listing (PVT Hx) with PVT at transplant (previously codified as PVT+, codified is this analysis as PVT Tx) on post-LT outcomes. Within the MASLD cohort, PVT Tx+ recipients, regardless of PVT history at listing (PVT Tx+ PVT Hx- and PVT Tx+ PVT Hx+), exhibited lower overall patient survival, graft survival, and death-censored graft survival rates compared to those PVT Tx− (Figure S6A,C,E). Similar trends were observed in the non-MASLD cohort (Figure S6B,D,F).

#### 3.3.3. Multivariate Analysis of Post-Transplant Outcomes

On multivariate analysis adjusted for recipient and donor characteristics (Tables S2–S4), we found that compared to patients without PVT, both MASLD and non-MASLD PVT+ showed poorer post-LT outcomes (Table 3). MASLD PVT+ had a significantly higher risk of all-cause mortality (adjusted hazard ratio [aHR] 1.55, 95% confidence interval [CI] 1.29–1.80, *p* < 0.001), graft failure (aHR 1.54, 95% CI 1.31–1.83, *p* < 0.001), and DCGF (aHR 1.50, 95% CI 1.12–2.01, *p* = 0.007). Similarly, non-MASLD PVT+ patients also had a significantly higher risk of all-cause mortality (aHR 1.56, 95% CI 1.39–1.76, *p* < 0.001), graft failure (aHR 1.50, 95% CI 1.36–1.67, *p* < 0.001), and DCGF (aHR 1.38, 95% CI 1.20–1.59, *p* < 0.001). Presence of PVT did not significantly affect post-LT outcomes between MASLD and non-MASLD recipients.

When stratified by sex within the MASLD cohort, the presence of PVT at the time of transplant in male recipients was significantly associated with higher risk of all-cause mortality (aHR 1.70, 95% CI 1.34–2.16, *p* < 0.001), graft failure (aHR 1.65, 95% CI 1.33–2.04, *p* < 0.001), and death-censored graft failure (aHR 1.50, 95% CI 1.06–2.14, *p* = 0.02). Among female recipients, no significant differences were observed in all-cause mortality (aHR 1.27, 95% CI 0.97–1.67, *p* = 0.08) or death-censored graft failure (aHR 1.42, 95% CI 0.91–2.22, *p* = 0.12), although PVT was significantly associated with graft failure (aHR 1.31, 95% CI 1.03–1.67, *p* = 0.03).

### 3.4. Effect of PVT on Post-Transplant Outcomes in Recipients of Livers from DCD Donors

**Figure 3:**
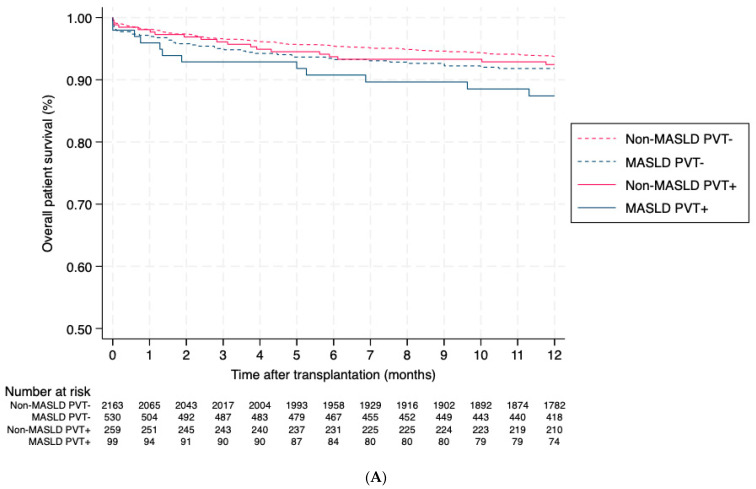

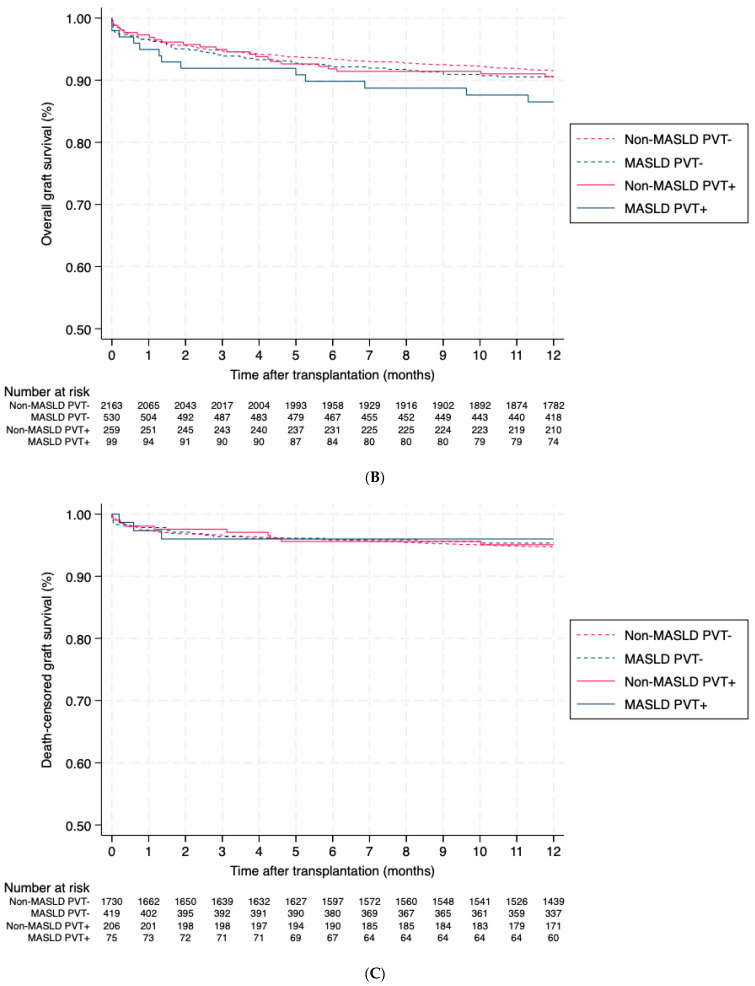
Kaplan–Meier curves of (**A**) overall patient survival, (**B**) graft survival and (**C**) death-censored graft survival after one-year post-LT in recipients of livers from donation after circulatory death donors. Abbreviations: LT, liver transplantation; MASLD, metabolic dysfunction-associated steatotic liver disease; PVT, portal vein thrombosis.

A total of 3051 recipients (6.5%) received grafts from DCD donors, among whom PVT at the time of transplant was present in 15.7% of MASLD recipients and 10.7% of non-MASLD recipients (*p* < 0.001). Kaplan–Meier analysis demonstrated significantly worse patient survival in MASLD PVT+ patients compared to non-MASLD PVT− (*p* = 0.013; Figure 3A). However, no significant differences were observed in graft survival or death-censored graft survival (Figure 3B and Figure 3C, respectively).

In multivariate analysis using non-MASLD PVT− recipients as the reference group, MASLD PVT+ had a significantly higher risk of all-cause mortality (aHR 2.24, 95% CI 1.17–4.28, *p* = 0.01). This finding is limited by the small simple size leading to imprecision of hazard ratio estimates. No significant differences were observed for graft failure (aHR 1.74, 95% CI 0.95–3.21, *p* = 0.08) or death-censored graft failure (aHR 1.05, 95% CI 0.31–3.50, *p* = 0.93) (Table 4).

In multivariate analysis restricted to MASLD recipients undergoing liver transplantation with DCD grafts, we observed that those with PVT at the time of transplant had a higher, though not statistically significant, risk of adverse outcomes compared to those without PVT. Specifically, PVT was associated with increased all-cause mortality (adjusted hazard ratio [aHR] 1.97; 95% CI, 0.98–3.96; *p* = 0.06), graft failure (aHR 1.81; 95% CI, 0.94–3.50; *p* = 0.08), and death-censored graft failure (aHR 1.16; 95% CI, 0.29–4.75; *p* = 0.22).

### 3.5. Causes of Death and Graft Failure in Study Cohort

A significant difference was observed in the causes of death within the first-year post-LT between MASLD and non-MASLD recipients (Table S5). The most common causes of death within one year were infection (23.5%) and cardiovascular complications (21.0%). Within the MASLD group, cardiovascular complications were the leading cause of death, particularly among recipients with PVT, in whom the incidence was higher compared to those without PVT (29.0% vs. 23.0%, *p* < 0.001). In contrast, among non-MASLD recipients, infection was the primary cause of death, with a significantly higher rate observed in those with PVT compared to those without PVT (24.7% vs. 23.0%, *p* < 0.001).

Primary non-function (37.4%) and infection (21.4%) were the most common causes of graft failure in our cohort (Table S6). Primary non-function was the predominant cause of graft failure in both MASLD and non-MASLD recipients, irrespective of PVT status. Graft failure resulting from hepatic artery thrombosis occurred more frequently in MASLD PVT+ than in MASLD PVT−.

## 4. Discussion

In this national retrospective cohort study of U.S. LT recipients, while we observed that the presence of PVT at the time of transplant was significantly associated with increased all-cause mortality, graft failure, and DCGF within the first-year post-transplantation in both MASLD and non-MASLD patients, the clinical outcomes did not differ significantly between these two groups. When restricting the analysis to only recipients receiving grafts from DCD donors, we found that MASLD PVT+ recipients exhibited the highest all-cause mortality. This is the first study to explore the effect of PVT in transplant outcomes comparing MASLD to non-MASLD patients and studying the effect of PVT in MASLD recipients undergoing transplantation from DCD donors.

Our study showed that PVT worsens post-transplant outcomes in both MASLD and non-MASLD; however, no significant changes were found between these two groups. While our findings are consistent with prior research which reported that PVT at time of LT in metabolic dysfunction-associated steatohepatitis (MASH) recipients increased the risk of graft failure and all-cause mortality [10], our study builds on these findings by leveraging a larger sample size, extending the observation period, and incorporating DCGF as a critical outcome measure. This approach allows for a more precise identification of the intrinsic factors contributing to graft failure, which might otherwise be obscured if death were not censored. Furthermore, we observed a steady increase in the prevalence of PVT among MASLD recipients over the past decade, rising from 12% to 17% in the most recent five-year period (2017–2022). Several factors likely contribute to this increasing prevalence. First, the parallel trends of worsening obesity and the MASLD epidemic have coincided with an increasing demand for LT. Second, advances in surgical techniques have made surgeons more adept and comfortable with performing transplants on patients with PVT [8]. Third, the established link between MASLD and PVT as a risk factor has led to heightened awareness and early detection of PVT in this patient population [18].

The presence of PVT at the time of transplant was associated with a higher risk of all-cause mortality in MASLD recipients who received grafts from DCD donors. Although prior studies have demonstrated that selected patients with low-grade PVT can safely undergo DCD liver transplantation without adversely affecting survival or graft function, current international guidelines do not recommend the routine use of DCD grafts in recipients with complex PVT due to increased technical difficulty and risk [13,19,20]. Some studies showed that while grade I–II PVT may be compatible with acceptable outcomes using DCD grafts, patients with grade III–IV PVT or uncharacterized thrombosis had significantly worse outcomes [20]. While the impact of donor steatosis on transplant outcomes has been previously studied, to our knowledge, no prior investigation has specifically evaluated the combined effect of PVT and DCD graft use in MASLD recipients. Our findings may reflect the complex interplay between the higher prevalence of obesity and diabetes in MASLD, and the increased perioperative risk associated with PVT. The addition of DCD grafts in this setting may further compound technical and hemodynamic challenges during transplantation, contributing to poorer outcomes in this high-risk subgroup.

When evaluating the association of PVT at listing and at the time of transplantation, our study found that MASLD recipients with PVT at LT, regardless of PVT status at listing, exhibited poorer post-LT outcomes. A similar trend was observed in the non-MASLD cohort, consistent with previous studies linking newly diagnosed PVT at LT to lower 90-day patient and graft survival compared to those without PVT at either listing or LT [21]. Despite concerns about the accuracy of PVT data in the OPTN database, Ghabril et al. highlighted distinct clinical phenotypes within these subgroups, reinforcing the importance of PVT status throughout the transplant process [21].

In addition, we observed a higher rate of cardiovascular-related death among MASLD recipients with PVT compared to those without PVT. This may reflect a greater burden of underlying cardiometabolic dysfunction in patients who develop PVT, factors that are not fully captured by conventional variables such as MELD score, age, or BMI. Emerging evidence suggests that patients with cardiometabolic disease may harbor distinct polygenic risk profiles that predispose them to venous thromboembolism, in some cases with a risk magnitude comparable to that of established monogenic thrombophilias [22]. It is therefore plausible that MASLD patients who develop PVT represent a genetically and phenotypically distinct subgroup with heightened prothrombotic and cardiovascular risk, ultimately contributing to poorer post-transplant outcomes.

The rates of hepatic artery thrombosis (HAT) were higher in MASLD patients with PVT (PVT+) compared to those without PVT (PVT−). In a large U.S. retrospective cohort study, pre-transplant PVT was associated with a two-fold increased risk of developing HAT, leading to graft loss within 90 days post-transplantation [23]. The study’s authors hypothesized that a combination of endothelial dysfunction, a predisposition to post-LT hypercoagulability, surgical techniques, and cold ischemia contribute to the development of HAT in patients with PVT. Additionally, the pro-inflammatory state and hypercoagulability commonly observed in MASLD may further exacerbate this risk, potentially explaining why HAT rates were higher in PVT+ MASLD patients compared to PVT+ non-MASLD patients.

To date, this is the largest study examining PVT in recipients with MASLD and, to our knowledge, the first to study the effect PVT in MASLD patients undergoing transplantation from DCD donors. However, our study has some limitations. First, its retrospective design may introduce selection bias. Second, the lack of granularity in the UNOS database limits the evaluation of factors influencing the development, treatment, and prognosis of PVT, such as inherited thrombophilia, anticoagulation use for other conditions, and detailed characterization of thrombosis, including extent, chronicity, and intraoperative management strategies, all of which may affect post-transplant outcomes [8]. Third, while we excluded patients with a prior history of TIPS due to the lack of granular data to characterize its specific effect on PVT in MASLD patients, future studies should explore whether outcomes differ by liver disease etiology among those with PVT who underwent TIPS. Fourth, although categorizing cryptogenic cirrhosis with BMI ≥ 30 kg/m^2^ as MASLD may introduce heterogeneity, previous studies have shown that including this subset of patients enhances the generalizability of findings [3,14,15,24,25]. Fifth, a key limitation of our study is the lack of data on graft preservation techniques, particularly ex situ machine perfusion, which is underreported in the UNOS STAR files. Hypothermic oxygenated machine perfusion (HOPE) has been shown to reduce biliary complications after DCD liver transplantation and may alter the risk profile in recipients with PVT [25]. Future studies should examine its role in this high-risk population. Finally, excluding HCC patients may have inadvertently excluded a substantial cohort of patients with low natural MELD scores who were transplanted using MELD exceptions.

In conclusion, we found that while the presence of PVT at time of transplantation was associated with poor transplant outcomes in both MASLD and non-MASLD recipients; no significant difference was found between the two groups. Within the DCD group, MASLD patients with PVT at time of transplantation had increased significant risk for all-cause mortality. Given the increase trends of MASLD as a transplant indication, the persistent organ shortage and increased utilization of DCD grafts, careful recipient selection should be implemented, particularly in patients with PVT at time of transplantation.

## Figures and Tables

**Table 1:** Baseline characteristics of study population.

	MASLD				Non-MASLD			
	Overall (*n* = 9362)	PVT+ (*n* = 1465)	PVT− (*n* = 7897)	*p*-Value	Overall (*n* = 37,631)	PVT+ (*n* = 3283)	PVT− (*n* = 34,348)	*p*-Value
Recipient characteristics								
Age ^†^, mean ± SD	58.1 ± 8.8	59.4 ± 7.8	57.9 ± 8.9	<0.01	52.2 ± 10.4	54.3 ± 10.1	52 ± 10.4	<0.01
Female sex, *n* (%)	4198 (48.1)	614 (45.0)	3584 (48.7)	0.03	12,534 (35.6)	1006 (33.3)	11,528 (35.8)	<0.01
Race/ethnicity, *n* (%)				0.03				<0.01
NH White	7066 (80.9)	1085 (79.4)	5981 (81.2)		25,712 (73)	2144 (70.9)	23,568 (73.2)	
NH Black	191 (2.2)	24 (1.8)	167 (2.3)		3221 (9.2)	232 (7.7)	2989 (9.3)	
Hispanic	1212 (13.9)	213 (15.6)	999 (13.6)		4741 (13.5)	512 (16.9)	4229 (13.1)	
NH Asian	135 (1.6)	21 (1.5)	114 (1.6)		1059 (3.0)	90 (3.0)	969 (3.0)	
Other	129 (1.5)	23 (1.7)	106 (1.4)		476 (1.4)	46 (1.5)	430 (1.3)	
Blood type, *n* (%)				0.79				0.86
Group O	3882 (44.5)	619 (45.3)	3263 (44.3)		15,622 (44.4)	1361 (45)	14,261 (44.3)	
Group A	3241 (37.1)	502 (36.8)	2739 (37.2)		12,900 (36.6)	1092 (36.1)	11,808 (36.7)	
Group B	1139 (13.0)	174 (12.7)	965 (13.1)		4743 (13.5)	406 (13.4)	4337 (13.5)	
Group AB	471 (5.4)	71 (5.2)	400 (5.4)		1944 (5.5)	165 (5.5)	1779 (5.5)	
BMI, kg/m^2^, mean ± SD	33.4 ± 5.9	33.4 ± 5.7	33.3 ± 5.9	0.77	28.4 ± 5.7	28.4 ± 5.4	28.4 ± 5.7	0.48
BMI ≥ 30 kg/m^2^, *n* (%)	6344 (72.6)	1012 (74.1)	5332 (72.4)	0.69	11,844 (33.6)	1034 (34.2)	10,810 (33.6)	0.57
Presence of diabetes, *n* (%)	4333 (49.6)	788 (57.7)	3545 (48.1)	<0.01	5822 (16.5)	665 (22.0)	5157 (16.0)	<0.01
Presence of encephalopathy, *n* (%)	6630 (75.9)	1067 (78.1)	5563 (75.5)	0.14	25,707 (73.0)	2248 (74.3)	23,459 (72.9)	0.20
Presence of ascites, *n* (%)	7722 (88.4)	1216 (89.0)	6506 (88.3)	0.96	30,504 (86.6)	2668 (88.2)	27,836 (86.5)	0.05
Presence of SBP, *n* (%)	801 (9.2)	150 (11.0)	651 (8.8)	0.01	3771 (10.7)	443 (14.7)	3328 (10.3)	<0.01
Prior dialysis ^‡^, *n* (%)	1099 (12.6)	152 (11.1)	947 (12.9)	<0.01	4430 (12.6)	366 (12.1)	4064 (12.6)	0.70
Prior abdominal surgery, *n* (%)	5313 (60.8)	868 (63.5)	4445 (60.3)	<0.01	15,746 (44.7)	1627 (53.8)	14,119 (43.9)	<0.01
MELD ^§^, median (IQR)	24 (19–31)	24 (19–30)	25 (19–31)	<0.01	25.9 (19–33)	25 (19–33)	25.9 (19–33)	<0.01
Serum albumin, g/dL, mean ± SD	3.1 ± 0.7	3.1 ± 0.7	3.1 ± 0.7	0.04	3.0 ± 0.7	3.1 ± 0.7	3.0 ± 0.7	<0.01
Serum bilirubin, mg/dL, median (IQR)	4.5 (2.5–9.3)	3.4 (1.9–7.1)	4.7 (2.6–9.7)	<0.01	6.9 (3.2–15.5)	5 (2.5–12.6)	7 (3.3–15.8)	<0.01
INR, median (IQR)	1.8 (1.5–2.3)	1.9 (1.5–2.4)	1.8 (1.5–2.3)	<0.01	1.9 (1.5–2.5)	1.9 (1.5–2.5)	1.9 (1.5–2.5)	0.79
Creatinine, mg/dL, median (IQR)	1.3 (0.9–1.9)	1.3 (1.0–1.9)	1.3 (0.9–1.9)	0.14	1.2 (0.8–1.8)	1.2 (0.9–1.8)	1.2 (0.8–1.8)	0.03
Sodium, mg/dL, mean ± SD	134.8 ± 5.0	135 ± 4.9	134.8 ± 5.1	0.04	134.6 ± 5.0	134.9 ± 5.2	134.6 ± 5.0	<0.01
History of PVT, *n* (%)	785 (8.9)	644 (47.1)	141 (2.0)	<0.01	1600 (4.5)	1160 (38.3)	440 (1.4)	<0.01
Cause of Liver disease, *n* (%)				0.999				<0.01
MASLD	8733 (100%)	1366 (15.6)	7367 (84.3)		-	-	-	
ALD	-	-	-		14,606 (41.4)	1141 (37.7)	13,465 (41.8)	
HBV	-	-	-		1178 (3.4)	89 (2.9)	1089 (3.4)	
HCV	-	-	-		9348 (26.5)	817 (27.0)	8531 (26.5)	
Autoimmune	-	-	-		7165 (20.3)	640 (21.2)	6525 (20.2)	
Other	-	-	-		2912 (8.2)	337 (11.1)	2575 (8.0)	
Transplant era, *n* (%)				<0.01				<0.01
2002–2006	970 (11.1)	85 (6.2)	885 (12.0)		9122 (25.9)	432 (14.3)	8690 (27.0)	
2007–2011	1430 (16.4)	195 (14.3)	1235 (16.8)		7051 (20.0)	646 (21.4)	6405 (19.9)	
2012–2016	1982 (22.7)	338 (24.7)	1644 (22.3)		6702 (19.0)	804 (26.6)	5898 (18.3)	
2017–2022	4351 (49.8)	748 (54.8)	3603 (48.9)		12,334 (35.0)	1142 (37.8)	11,192 (34.8)	
Geographic region, *n* (%)				0.04				<0.01
Northeast	1315 (15.1)	173 (12.7)	1142 (15.5)		7233 (20.5)	481 (15.9)	6752 (21.0)	
Southeast	3856 (44.2)	622 (45.5)	3234 (43.9)		13,862 (39.4)	1196 (39.6)	12,666 (39.4)	
Midwest	2320 (26.6)	381 (27.9)	1939 (26.3)		8216 (23.3)	759 (25.1)	7457 (23.2)	
West	1242 (14.2)	190 (13.9)	1052 (14.3)		5898 (16.8)	588 (19.4)	5310 (16.5)	
Donor characteristics								
Donor age mean ± SD ^†^	42.9 ± 15.8	43.1 ± 15.5	42.9 ± 15.8	0.16	41.4 ± 15.6	41.9 ± 15.4	41.3 ± 15.7	0.01
Donor sex, female *n* (%)	5248 (60.1)	820 (60.0)	4428 (60.1)	0.79	21,138 (60.0)	1828 (60.5)	19,310 (60.0)	0.75
Donor race/ethnicity, *n* (%)				0.04				<0.01
NH White	5770 (66.1)	888 (65.0)	4882 (66.3)		23,281 (66.1)	1968 (65.1)	21,313 (66.2)	
NH Black	1630 (18.7)	271 (19.8)	1359 (18.5)		6027 (17.1)	543 (18.0)	5484 (17.0)	
Hispanic	1054 (12.1)	172 (12.6)	882 (12.0)		4832 (13.7)	411 (13.6)	4421 (13.7)	
NH Asian	194 (2.2)	30 (2.2)	164 (2.2)		722 (2.1)	64 (2.1)	658 (2.0)	
Other	85 (1.0)	5 (0.4)	80 (1.1)		347 (1.0)	38 (1.3)	309 (1.0)	
Donor BMI, kg/m^2^, median (IQR)	28.3 ± 6.5	28.4 ± 6.5	28.3 ± 6.5	<0.01	27.5 ± 6.1	27.9 ± 6.3	27.5 ± 6.1	<0.01
Donor BMI ≥ 30, *n* (%)	2896 (33.2)	459 (33.6)	2347 (33.1)	0.94	9947 (28.3)	911 (30.1)	9036 (28.1)	<0.01
Diabetes, *n* (%)	1161 (13.3)	195 (14.3)	966 (13.1)	0.27	3950 (11.2)	357 (11.8)	3593 (11.2)	0.07
Cold ischemia time, hours, mean ± SD	6.3 ± 2.4	6.4 ± 2.3	6.2 ± 2.4	<0.01	6.5 ± 2.4	6.6 ± 2.4	6.5 ± 2.4	<0.01
DRI, median (IQR)	1.4 (1.2–2.2)	1.4 (1.2–2.2)	1.4 (1.2–2.2)	0.20	1.3 (1.1–1.8)	1.6 (1.1–1.9)	1.3 (1.1–1.8)	0.44
DRI categories ^¶^, *n* (%)				0.25				0.50
Low-DRI	4557 (52.2)	696 (51.0)	3861 (52.4)		19,573 (55.6)	1653 (54.7)	17,920 (55.7)	
Medium-DRI	1739 (19.9)	270 (19.8)	1469 (19.9)		7040 (20.0)	615 (20.3)	6425 (20.0)	
High-DRI	2437 (27.9)	400 (29.3)	2037 (27.7)		8596 (24.4)	756 (25.0)	7840 (24.4)	
Graft type, *n* (%)				0.95				<0.001
DBD	37,631 (93.3)	1366 (93.2)	7367 (93.3)		34,348 (91.3)	3024 (92.1)	32,185 (93.7)	
DCD	629 (6.7)	99 (6.8)	530 (6.7)		3283 (8.7)	259 (7.9)	2163 (6.3)	
Donor–recipient match per BSA, *n* (%)				0.85				0.24
Too small	303 (3.5)	50 (3.7)	253 (3.4)		676 (1.9)	57 (1.9)	619 (1.9)	
Appropriate size	8048 (92.2)	1253 (91.7)	6795 (92.2)		32,207 (91.5)	2749 (90.9)	29,458 (91.5)	
Too large	382 (4.4)	63 (4.6)	319 (4.3)		2326 (6.6)	218 (7.2)	2108 (6.6)	

^†^ Recipient and donor age in years. ^‡^ Most recent waiting list dialysis twice in a prior week before transplant or at removal if removed. ^§^ At the time of transplant. ^¶^ DRI categorized using tertiles. Abbreviations: DBD, donation after brain death; DCD, donation after circulatory death; MASLD, metabolic dysfunction-associated steatotic liver disease; PVT, portal vein thrombosis; SD, standard deviation; NH, non-Hispanic; BMI, body mass index; SBP, spontaneous bacterial peritonitis; MELD, Model for End-Stage Liver Disease; IQR (inter-quartile range); INR, international normalized ratio; ALD, alcoholic liver disease; HBV, hepatitis B; HCV, hepatitis C; DRI, donor risk index; BSA, body surface area.

**Table 2:** Survival rates of PVT+ and PVT− MASLD and non-MASLD recipients.

	MASLD (*n* = 9362)			Non-MASLD (*n* = 37,631)		
	Patient Survival, 95% CI	Graft Survival, 95% CI	Death-Censored Graft Survival, 95% CI	Patient Survival, 95% CI	Graft Survival, 95% CI	Death-Censored Graft Survival, 95% CI
1 month						
PVT+	95.8 (94.6–96.8)	94.3 (92.9–95.4)	96.42 (95.1–97.4)	96.3 (95.6–96.9)	94.3 (93.4–95.1)	96.16 (95.3–96.9)
PVT−	97.7 (97.3–98.0)	96.8 (96.4–97.2)	98.13 (97.7–98.4)	97.6 (97.5–97.8)	96.3 (96.1–96.5)	97.44 (97.2–97.6)
3 months						
PVT+	93.3 (91.8–94.5)	91.5 (89.8–92.8)	96.04 (94.7–97.1)	93.3 (92.3–94.2)	91.0 (89.9–91.9)	94.67 (93.7–95.5)
PVT−	95.6 (95.1–96.1)	94.4 (93.8–94.9)	97.21 (96.7–97.6)	95.9 (95.6–96.1)	94.0 (93.7–94.3)	96.27 (96.0–96.5)
6 months						
PVT+	91.4 (89.7–92.8)	89.2 (87.4–90.8)	95.18 (93.7–96.3)	91.5 (90.4–92.4)	88.6 (87.5–89.7)	93.51 (92.4–94.4)
PVT−	94.1 (93.5–94.6)	92.5 (91.9–93.1)	96.55 (96.0–97.0)	94.2 (93.9–94.4)	92.0 (91.7–92.3)	95.13 (94.8–95.4)
1 year						
PVT+	88.9 (87.1–90.5)	86.5 (84.5–88.2)	94.15 (92.5–95.4)	88.9 (87.7–90.0)	85.8 (84.5–87.0)	92.15 (91.0–93.2)
PVT−	92.1 (91.5–92.7)	90.3 (89.6–90.9)	95.56 (95.0–96.1)	91.8 (91.5–92.1)	89.0 (88.7–89.4)	93.30 (93.0–93.6)

Abbreviations: CI, confidence interval; MASLD, metabolic dysfunction-associated liver disease; PVT, portal vein thrombosis.

**Table 3:** Multivariate Cox regression analysis of the association between PVT at time of transplant and post-transplant outcomes in the patient cohort.

	MASLD			Non-MASLD		
PVT+ (Ref. PVT−)	aHR	95% CI	*p*-Value	aHR	95% CI	*p*-Value
All-cause mortality ^†^	1.55	1.29–1.87	<0.001	1.56	1.39–1.76	<0.001
Graft failure ^†^	1.54	1.31–1.83	<0.001	1.50	1.36–1.67	<0.001
DCGF ^†^	1.50	1.12–2.01	0.007	1.38	1.20–1.59	<0.001

^†^ Within the non-MASLD cohort, we performed a statistical analysis using “strata( )” function in STATA to control for diabetes and obesity on multivariate analysis to avoid violation of the proportionality hazard assumption. Abbreviations: aHR, adjusted hazard ratio; CI, confidence interval; DCGF, death-censored graft failure; MASLD, metabolic dysfunction-associated steatotic liver disease; PVT, portal vein thrombosis.

**Table 4:** Multivariate Cox regression analysis of the association between PVT at time of transplant and post–transplant outcomes in recipients of livers from donation after circulatory death donors.

	All-Cause Mortality ^†^			Graft Failure ^†^			Death-Censored Graft Failure ^†^		
Etiology	aHR	95% CI	*p*-Value	aHR	95% CI	*p*-Value	aHR	95% CI	*p*-Value
**Non-MASLD PVT− as the reference**									
Non-MASLD PVT−	1.00			1.00			1.00		
MASLD PVT−	1.38	0.92–2.10	0.11	1.12	0.77–1.61	0.56	1.01	0.57–1.80	0.96
Non-MASLD PVT+	1.37	0.83–2.24	0.21	1.26	0.82–1.96	0.30	1.10	0.56–2.16	0.77
MASLD PVT+	2.24	1.17–4.28	0.01	1.74	0.95–3.21	0.08	1.05	0.31–3.50	0.93
**MASLD PVT− as the reference**									
MASLD PVT−	1.00			1.00			1.00		
MASLD PVT+	1.97	0.98–3.96	0.06	1.81	0.94–3.50	0.08	1.16	0.29–4.75	0.22

^†^ Within the non-MASLD cohort we performed a statistical analysis using “strata( )” function in STATA to control for diabetes and obesity on multivariate analysis to avoid violation of the proportionality hazard assumption. Abbreviations: aHR, adjusted hazard ratio; CI, confidence interval; DCGF, death-censored graft failure; MASLD, metabolic dysfunction-associated steatotic liver disease; PVT, portal vein thrombosis.

## Data Availability

Data can be requested at https://unos.org/data/ (accessed on 30 June 2023).

## Supplementary Materials

The following supporting information can be downloaded at: https://www.mdpi.com/article/10.3390/jcm15051787/s1, Table S1: Diagnosis codes for etiologies included in patient cohort; Table S2: Univariate and multivariate Cox regression analysis of all-cause mortality in deceased-donor liver transplant MASLD and non-MASLD recipients between 2002 to 2022 in the U.S.; Table S3: Univariate and multivariate Cox regression analysis of graft-failure in deceased-donor liver transplant MASLD and non-MASLD recipients between 2002 to 2022 in the U.S.; Table S4: Univariate and multivariate Cox regression analysis of death-censored graft-failure in deceased-donor liver transplant MASLD and non-MASLD recipients between 2002 to 2022 in the U.S.; Table S5: Causes of death within the first year after liver transplantations in patients with MASLD and Non-MASLD, stratified by PVT status; Table S6: Causes of graft failure in recipients with MASLD and Non-MASLD undergoing transplantation, stratified by PVT status; Figure S1: Prevalence of PVT in LT recipients across 5-year intervals periods. (A) MASLD and non-MASLD. (B) MASLD and non-MASLD stratified by etiologies; Figure S2: Kaplan–Meier curves of patient survival after one-year post-LT in patients with MASLD and Non-MASLD, stratified by presence or absence of PVT across 5-year interval periods: (A) 2002–2006 (B) 2007–2011 (C) 2012–2016 (D) 2017–2022; Figure S3: Kaplan–Meier curves of graft survival after one-year post-LT in patients with MASLD and Non-MASLD, stratified by presence or absence of PVT across 5-year interval periods: (A) 2002–2006 (B) 2007–2011 (C) 2012–2016 (D) 2017–2022; Figure S4: Kaplan–Meier curves of death-censored graft survival after one-year post-LT in patients with MASLD and Non-MASLD, stratified by presence or absence of PVT across 5-year interval periods: (A) 2002–2006 (B) 2007–2011 (C) 2012–2016 (D) 2017–2022; Figure S5: Kaplan–Meier curves of (A) overall patient survival, (B) graft survival and (C) death-censored graft survival after LT in patients with PVT at time of transplant, stratified by etiology of liver disease; Figure S6: Kaplan–Meier curves of overall patient survival (A, B), graft survival (C, D) and death-censored graft survival (E, F) after one-year post-LT in patients with MASLD and non-MASLD with and without PVT at listing (PVT Hx) and at transplant (PVT Tx).

## Abbreviations

The following abbreviations are used in this manuscript: AILDs, autoimmune liver diseases; aHR, adjusted hazard ratio; ALD, alcoholic liver disease; BMI, body mass index; BSA, body surface area; CI, confidence interval; CIT, cold ischemia time; DAA, direct-acting antiviral; DBD, donation after brain death; DCD, donation after circulatory death; DCGF, death-censored graft failure; DRI, donor risk index; HAT, hepatic artery thrombosis; HBV, hepatitis B virus; HCC, hepatocellular carcinoma; HCV, hepatitis C virus; HR, hazard ratio; INR, international normalized ratio; IQR, interquartile range; LT, liver transplantation; MASLD, metabolic dysfunction-associated steatotic liver disease; MASH, metabolic dysfunction-associated steatohepatitis; MELD, Model for End-Stage Liver Disease; OPTN, Organ Procurement and Transplantation Network; PVT, portal vein thrombosis; PVT Hx, history of portal vein thrombosis at listing; PVT Tx, portal vein thrombosis at time of transplant; SBP, spontaneous bacterial peritonitis; SD, standard deviation; STAR, Standard Transplant Analysis and Research; TCR, Transplant Candidate Report; TIPS, transjugular intrahepatic portosystemic shunt; TRR, Transplant Recipient Report; UNOS, United Network for Organ Sharing.

## References

[1] Miao L., Targher G., Byrne C.D., Cao Y.-Y., Zheng M.-H. (2024). Current Status and Future Trends of the Global Burden of MASLD. Trends Endocrinol. Metab..

[2] Younossi Z.M., Stepanova M., Ong J., Trimble G., AlQahtani S., Younossi I., Ahmed A., Racila A., Henry L. (2021). Nonalcoholic Steatohepatitis Is the Most Rapidly Increasing Indication for Liver Transplantation in the United States. Clin. Gastroenterol. Hepatol..

[3] Haldar D., Kern B., Hodson J., Armstrong M.J., Adam R., Berlakovich G., Fritz J., Feurstein B., Popp W., Karam V. (2019). Outcomes of Liver Transplantation for Non-Alcoholic Steatohepatitis: A European Liver Transplant Registry Study. J. Hepatol..

[4] Tripodi A., Fracanzani A.L., Primignani M., Chantarangkul V., Clerici M., Mannucci P.M., Peyvandi F., Bertelli C., Valenti L., Fargion S. (2014). Procoagulant Imbalance in Patients with Non-Alcoholic Fatty Liver Disease. J. Hepatol..

[5] Papatheodoridis G.V., Chrysanthos N., Cholongitas E., Pavlou E., Apergis G., Tiniakos D.G., Andrioti E., Theodossiades G., Archimandritis A.J. (2009). Thrombotic Risk Factors and Liver Histologic Lesions in Non-Alcoholic Fatty Liver Disease. J. Hepatol..

[6] Montenovo M., Rahnemai-Azar A., Reyes J., Perkins J. (2018). Clinical Impact and Risk Factors of Portal Vein Thrombosis for Patients on Wait List for Liver Transplant. Exp. Clin. Transplant..

[7] Ayala R., Grande S., Bustelos R., Ribera C., García-Sesma A., Jimenez C., Moreno E., Martínez-López J. (2012). Obesity Is an Independent Risk Factor for Pre-Transplant Portal Vein Thrombosis in Liver Recipients. BMC Gastroenterol..

[8] Ponziani F.R., Zocco M.A., Senzolo M., Pompili M., Gasbarrini A., Avolio A.W. (2014). Portal Vein Thrombosis and Liver Transplantation: Implications for Waiting List Period, Surgical Approach, Early and Late Follow-Up. Transplant. Rev..

[9] Zanetto A., Rodriguez-Kastro K.-I., Germani G., Ferrarese A., Cillo U., Burra P., Senzolo M. (2018). Mortality in Liver Transplant Recipients with Portal Vein Thrombosis—An Updated Meta-Analysis. Transpl. Int..

[10] Agbim U., Jiang Y., Kedia S.K., Singal A.K., Ahmed A., Bhamidimarri K.R., Bernstein D.E., Harrison S.A., Younossi Z.M., Satapathy S.K. (2019). Impact of Nonmalignant Portal Vein Thrombosis in Transplant Recipients with Nonalcoholic Steatohepatitis. Liver Transpl..

[11] Stine J.G., Shah N.L., Argo C.K., Pelletier S.J., Caldwell S.H., Northup P.G. (2015). Increased Risk of Portal Vein Thrombosis in Patients with Cirrhosis Due to Nonalcoholic Steatohepatitis. Liver Transpl..

[12] Kim S.C., Foley D.P. (2024). Strategies to Improve the Utilization and Function of DCD Livers. Transplantation.

[13] Schlegel A., Foley D.P., Savier E., Flores Carvalho M., De Carlis L., Heaton N., Taner C.B. (2021). Recommendations for Donor and Recipient Selection and Risk Prediction: Working Group Report from the ILTS Consensus Conference in DCD Liver Transplantation. Transplantation.

[14] Ochoa-Allemant P., Trivedi H.D., Saberi B., Bonder A., Fricker Z.P. (2023). Waitlist and Posttransplantation Outcomes of Lean Individuals with Nonalcoholic Fatty Liver Disease. Liver Transpl..

[15] Wong R.J., Aguilar M., Cheung R., Perumpail R.B., Harrison S.A., Younossi Z.M., Ahmed A. (2015). Nonalcoholic Steatohepatitis Is the Second Leading Etiology of Liver Disease among Adults Awaiting Liver Transplantation in the United States. Gastroenterology.

[16] Lan K.K., Rosenberger W.F., Lachin J.M. (1995). Sequential Monitoring of Survival Data with the Wilcoxon Statistic. Biometrics.

[17] Hertz-Picciotto I., Rockhill B. (1997). Validity and Efficiency of Approximation Methods for Tied Survival Times in Cox Regression. Biometrics.

[18] Gong H., Zhong H., Xu H.-M., Liu X.-C., Li L.-P., Zhang D.-K. (2023). Insight into Increased Risk of Portal Vein Thrombosis in Nonalcoholic Fatty Liver Disease. Eur. J. Intern. Med..

[19] Zhang C., Calderon E., Chang Y.-H., Lu P., Durant A.M., Villa E.L., Katariya N.N., Jadlowiec C., Reddy K.S., Moss A. (2024). Portal Vein Thrombosis and Donation after Cardiac Death Liver Transplantation: Pre-Perfusion Data Implications for the Perfusion Era. Am. J. Surg..

[20] Mercado L.A., Bhangu H.K., Calderon E., Mathur A.K., Aqel B., Musto K.R., Watt K.D., Rosen C.B., Bolan C., LeGout J.D. (2022). DCD Liver Grafts Can Safely Be Used for Recipients with Grade I–II Portal Vein Thrombosis: A Multicenter Analysis. Transplant. Direct.

[21] Ghabril M., Agarwal S., Lacerda M., Chalasani N., Kwo P., Tector A.J. (2016). Portal Vein Thrombosis Is a Risk Factor for Poor Early Outcomes After Liver Transplantation: Analysis of Risk Factors and Outcomes for Portal Vein Thrombosis in Waitlisted Patients. Transplantation.

[22] Marston N.A., Melloni G.E.M., Gurmu Y., Bonaca M.P., Kamanu F.K., Roselli C., Lee C., Cavallari I., Giugliano R.P., Scirica B.M. (2021). Genetic Risk Score to Identify Risk of Venous Thromboembolism in Patients with Cardiometabolic Disease. Circ. Genom. Precis. Med..

[23] Stine J.G., Pelletier S.J., Schmitt T.M., Porte R.J., Northup P.G. (2016). Pre-Transplant Portal Vein Thrombosis Is an Independent Risk Factor for Graft Loss Due to Hepatic Artery Thrombosis in Liver Transplant Recipients. HPB.

[24] Medina-Morales E., Ismail M., Goyal R.M., Marenco-Flores A., Saberi B., Fricker Z., Bonder A., Trivedi H.D. (2024). Waitlist and Transplant Outcomes in Patients with Metabolic Dysfunction-Associated Steatotic Liver Disease and Autoimmune Hepatitis. Liver Int..

[25] van Rijn R., Schurink I.J., de Vries Y., van den Berg A.P., Cortes Cerisuelo M., Darwish Murad S., Erdmann J.I., Gilbo N., de Haas R.J., Heaton N. (2021). Hypothermic Machine Perfusion in Liver Transplantation—A Randomized Trial. N. Engl. J. Med..

