# Supplementary material for: Impact of Non-Malignant Portal Vein Thrombosis in Recipients with Metabolic Dysfunction-Associated Steatotic Liver Disease Compared to Other Transplant Indications
Source: J Clin Med. 2026 Feb 27;15(5):1787. doi: 10.3390/jcm15051787 (PMC12985707; doi:10.3390/jcm15051787)

SUPPLEMENTAL DIGITAL CONTENT

**TABLE S1.** Diagnosis codes for etiologies included in patient cohort	Page 2
**TABLE S2.** Univariate and multivariate Cox regression analysis of all-cause mortality in deceased-donor liver transplant MASLD and non-MASLD recipients between 2002 to 2022 in the U.S.	Pages 3-6
**TABLE S3.** Univariate and multivariate Cox regression analysis of graft-failure in deceased-donor liver transplant MASLD and non-MASLD recipients between 2002 to 2022 in the U.S.	Pages 7-10
**TABLE S4.** Univariate and multivariate Cox regression analysis of death-censored graft-failure in deceased-donor liver transplant MASLD and non-MASLD recipients between 2002 to 2022 in the U.S.	Pages 11-14
**TABLE S5.** Causes of death within the first year after liver transplantations in patients with MASLD and Non-MASLD, stratified by PVT status	Page 15
**TABLE S6.** Causes of graft failure in recipients with MASLD and Non-MASLD undergoing transplantation, stratified by PVT status	Page 16
**Figure S1.** Prevalence of PVT in LT recipients across 5-year intervals periods. (A) MASLD and non-MASLD. (B) MASLD and non-MASLD stratified by etiologies.	Page 17
**Figure S2.** Kaplan–Meier curves of patient survival after one-year post-LT in patients with MASLD and Non-MASLD, stratified by presence or absence of PVT across 5-year interval periods: (A) 2002-2006 (B) 2007-2011 (C) 2012-2016 (D) 2017-2022.	Page 18
**Figure S3**. Kaplan–Meier curves of graft survival after one-year post-LT in patients with MASLD and Non-MASLD, stratified by presence or absence of PVT across 5-year interval periods: (A) 2002-2006 (B) 2007-2011 (C) 2012-2016 (D) 2017-2022.	Page 19
**Figure S4.** Kaplan–Meier curves of death-censored graft survival after one-year post-LT in patients with MASLD and Non-MASLD, stratified by presence or absence of PVT across 5-year interval periods: (A) 2002-2006 (B) 2007-2011 (C) 2012-2016 (D) 2017-2022.	Page 20
**Figure S5.** Kaplan–Meier curves of (A) overall patient survival, (B) graft survival and (C) death-censored graft survival after LT in patients with PVT at time of transplant, stratified by etiology of liver disease.	Page 21
**Figure S6.** Kaplan–Meier curves of overall patient survival (A, B), graft survival (C, D) and death-censored graft survival (E, F) after one-year post-LT in patients with MASLD and non-MASLD with and without PVT at listing (PVT Hx) and at transplant (PVT Tx).	Page 22

Table S1. Diagnosis codes for etiologies included in patient cohort

Etiology	Diagnostic codification
**MASLD**	Diagnosis code 4214
**ALD**	Diagnosis codes 4215, 4216, and 4219 excluding acute alcoholic hepatitis
**AILDs**	PBC [diagnosis code 4220], PSC [diagnosis code 4240], and AIH [diagnosis code 4212]
**HCV**	Positive HCV serologic status or diagnosis codes 4204, 4206, and 4216
**HBV**	Either positive hepatitis B surface antigen, positive nucleic acid testing or diagnosis codes 4202, 4206, 4207, and 4592
**Other**	Diagnosis codes 4208 and 4213 for cryptogenic with BMI < 30 mg/kg², diagnosis code 4301 for Wilson's disease, diagnosis code 4302 for Hemochromatosis, and 4300 for Alpha-1 Antitrypsin deficiency

Abbreviations: ALD, alcoholic liver disease; AILDs, autoimmune liver diseases; AIH, autoimmune hepatitis; BMI, body mass index; HCV, hepatitis C; HBV, hepatitis B; PBC, primary biliary cholangitis; PSC, primary sclerosing cholangitis.

TABLE S2. Univariate and multivariate Cox regression analysis of all-cause mortality in deceased-donor liver transplant MASLD and non-MASLD recipients between 2002 to 2022 in the U.S.

	All-cause mortality											
	UNIVARIATE						MULTIVARIATE					
	MASLD			Non-MASLD			MASLD[b]			Non-MASLD[c]		
	aHR	95% CI	p-value	aHR	95% CI	p-value	aHR	95% CI	p-value	aHR	95% CI	p-value
**Recipient characteristics**												
PVT + (ref. PVT-)	1.44	1.20-1.72	<0.001	1.39	1.24-1.56	<0.001	1.55	1.29-1.87	<0.001	1.56	1.39-1.76	<0.001
Recipient age[a]	1.35	1.23-1.48	<0.001	1.32	1.27-1.38	<0.001	1.36	1.23-1.50	<0.001	1.33	1.28-1.39	<0.001
Female sex	1.15	0.99-1.33	0.07	1.10	1.02-1.19	0.01				1.05	0.97-1.14	0.22
Race (ref. NH White)												
Black	0.78	0.44-1.38	0.39	1.30	1.16-1.46	<0.001				1.29	1.15-1.46	<0.001
Hispanic	1.05	0.85-1.30	0.62	0.97	0.87-1.08	0.57				0.95	0.85-1.07	0.38
Asian	0.46	0.19-1.10	0.08	1.05	0.85-1.30	0.65				0.98	0.78-1.22	0.83
Other	0.95	0.51-1.77	0.87	1.10	0.80-1.49	0.56				1.25	0.92-1.71	0.16
Blood type (ref. O)												
A	1.11	0.94-1.31	0.21	0.97	0.89-1.05	0.47						
B	1.19	0.95-1.49	0.12	0.91	0.81-1.03	0.12						
AB	0.83	0.57-1.21	0.33	0.92	0.78-1.09	0.33						

BMI	0.99	0.98-1.01	0.31	1.00	1.00-1.01	0.24	0.99	0.98-1.01	0.28			
BMI ≥ 30	0.99	0.84-1.17	0.90	1.03	0.95-1.11	0.45						
Diabetes	1.29	1.11-1.49	0.001	1.44	1.32-1.57	<0.001	1.22	1.05-1.42	0.01			
Encephalopathy	1.27	1.06-1.53	0.01	1.39	1.27-1.52	<0.001	1.18	0.98-1.42	0.08	1.21	1.10-1.32	<0.001
Ascites	1.01	0.80-1.28	0.92	1.33	1.18-1.50	<0.001						
SBP	1.19	0.94-1.51	0.15	1.04	0.92-1.17	0.52						
Dialysis	1.66	1.38-2.01	<0.001	1.69	1.54-1.85	<0.001	1.24	0.98-1.56	0.06	1.44	1.28-1.62	<0.001
Previous abdominal surgery	1.18	1.01-1.38	0.03	1.16	1.07-1.24	<0.001				1.12	1.04-1.21	0.003
MELD[d]	1.02	1.01-1.03	<0.001	1.02	1.01-1.02	<0.001	1.02	1.01-1.03	<0.001	1.03	1.02-1.04	<0.001
Serum albumin	0.97	0.87-1.08	0.54	0.92	0.87-0.97	0.002				0.93	0.88-0.98	0.01
Serum bilirubin	1.01	1.01-1.02	<0.001	1.01	1.00-1.01	<0.001						
INR	1.01	0.93-1.10	0.78	1.04	1.01-1.08	0.01				0.97	0.93-1.01	0.19
Creatinine	1.19	1.13-1.25	<0.001	1.17	1.14-1.20	<0.001	1.09	1.02-1.17	0.01	1.03	0.99-1.06	0.11
Sodium	1.03	1.01-1.04	<0.001	1.03	1.02-1.04	<0.001	1.02	1.01-1.04	0.01			
History of PVT (ref. PVT+)												
History of PVT-	1.31	1.04-1.65	0.02	1.26	1.07-1.48	0.01						
Unknown	1.21	0.62-2.33	0.58	1.53	1.25-1.87	<0.001						

Transplant year, (ref. 2002-2006)												
2007-2011	0.73	0.57-0.93	0.01	0.85	0.77-0.94	0.001	0.66	0.52-0.85	0.001	0.72	0.65-0.80	<0.001
2012-2016	0.64	0.51-0.81	<0.001	0.60	0.53-0.66	<0.001	0.55	0.43-0.70	<0.001	0.47	0.42-0.52	<0.001
2017-2022	0.50	0.41-0.62	<0.001	0.44	0.39-0.48	<0.001	0.45	0.36-0.57	<0.001	0.37	0.33-0.41	<0.001
Geographic region, (ref. Northeast)												
Southeast	0.75	0.61-0.92	0.01	0.75	0.68-0.82	<0.001				0.87	0.79-0.96	0.01
Midwest	0.87	0.70-1.09	0.23	0.69	0.62-0.77	<0.001				0.78	0.70-0.87	<0.001
West	0.77	0.59-1.01	0.06	0.74	0.66-0.83	<0.001				0.73	0.65-0.82	<0.001
**Donor characteristics**												
Age[a]	1.05	1.00-1.10	0.03	1.08	1.06-1.11	<0.001	1.07	1.02-1.12	0.01	1.04	1.01-1.09	0.03
Sex, female	1.19	1.02-1.38	0.02	1.03	0.96-1.11	0.41						
Race/ethnicity (ref. NH White)												
NH Black	0.93	0.76-1.14	0.49	1.03	0.93-1.14	0.56						
Hispanic	1.28	1.03-1.58	0.02	1.09	0.98-1.22	0.10						
NH Asian	0.92	0.54-1.56	0.75	1.24	0.98-1.58	0.08						
Other	0.44	0.14-1.36	0.15	0.96	0.65-1.42	0.84						
BMI	1.00	0.99-1.01	0.93	1.00	0.99-1.00	0.28						

BMI ≥ 30	0.97	0.83-1.14	0.72	0.93	0.85-1.01	0.08						
Diabetes	1.22	1.00-1.50	0.05	1.14	1.02-1.27	0.02						
CIT	1.06	1.03-1.09	<0.001	1.07	1.05-1.08	<0.001	1.04	1.01-1.07	0.02	1.00	0.99-1.01	0.91
DRI	1.15	1.04-1.27	0.005	1.01	1.00-1.01	<0.001						
DRI categories (ref. Low-DRI)												
Medium-DRI	1.37	1.14-1.65	0.001	1.22	1.10-1.34	<0.001				1.12	1.00-1.25	0.04
High-DRI	1.23	1.03-1.46	0.02	1.35	1.24-1.47	<0.001				1.17	1.01-1.34	0.03
DCD (ref. DBD)	1.07	0.81-1.41	0.629	0.75	0.63-0.88	0.001	1.13	0.86-1.50	0.37	0.77	0.65-0.90	0.002
Donor-recipient match per BSA (ref. too small)												
Appropriate size	0.94	0.63-1.40	0.77	0.78	0.61-0.99	0.05				0.78	0.61-1.00	0.05
Too large	1.07	0.64-1.79	0.80	0.94	0.71-1.24	0.65				0.97	0.73-1.29	0.83

[a] Recipient and donor age divided by ten-years period.
[b] We stratified by gender on multivariate analysis to avoid violation of the proportionality hazard assumption.
[c] We stratified by diabetes and obesity on multivariate analysis to avoid violation of the proportionality hazard assumption.
[d] At the time of transplant.

Abbreviations: DBD, donation after brain death; DCD, donation after circulatory death MASLD, metabolic dysfunction-associated steatotic liver disease; PVT, portal vein thrombosis; SD, standard deviation; NH, non-Hispanic; BMI, body mass index; SBP, spontaneous bacterial peritonitis; MELD, Model for End-Stage Liver Disease; IQR (inter-quartile range); INR, international normalized ratio; DRI, donor risk index; BSA, body surface area; CIT, cold ischemia time.

TABLE S3. Univariate and multivariate Cox regression analysis of graft-failure in deceased-donor liver transplant MASLD and non-MASLD recipients between 2002 to 2022 in the U.S.

	Graft failure											
	UNIVARIATE						MULTIVARIATE					
	MASLD			Non-MASLD			MASLD			Non-MASLD[b]		
	aHR	95% CI	p-value	aHR	95% CI	p-value	aHR	95% CI	p-value	aHR	95% CI	p-value
**Recipient characteristics**												
PVT + (ref. PVT-)	1.42	1.21-1.68	<0.001	1.33	1.21-1.48	<0.001	1.54	1.31-1.83	<0.001	1.50	1.36-1.67	<0.001
Age[a]	1.18	1.09-1.28	<0.001	1.15	1.12-1.19	<0.001	1.19	1.09-1.29	<0.001	1.15	1.11-1.18	<0.001
Female sex	1.04	0.91-1.19	0.55	1.02	0.96-1.09	0.53	1.03	0.90-1.18	0.70	0.96	0.90-1.03	0.26
Race (ref. NH White)												
Black	0.83	0.51-1.36	0.46	1.27	1.15-1.41	<0.001				1.25	1.13-1.39	<0.001
Hispanic	1.00	0.82-1.21	0.98	0.94	0.85-1.03	0.20				0.92	0.84-1.02	0.11
Asian	0.73	0.39-1.36	0.32	1.00	0.83-1.21	0.98				0.94	0.78-1.14	0.53
Other	0.99	0.57-1.71	0.96	0.97	0.73-1.28	0.84				1.11	0.84-1.47	0.47
Blood type (ref. O)												
A	1.08	0.93-1.26	0.29	0.95	0.88-1.02	0.14						
B	1.15	0.94-1.40	0.19	0.92	0.83-1.02	0.10						
AB	0.92	0.67-1.27	0.61	0.97	0.85-1.12	0.72						

BMI	1.00	0.99-1.01	0.82	1.00	1.00-1.01	0.17	1.00	0.98-1.01	0.53	1.00	0.98-1.02	0.79
BMI ≥ 30	1.10	0.95-1.28	0.21	1.03	0.97-1.10	0.36						
Diabetes	1.24	1.08-1.41	0.002	1.30	1.20-1.40	<0.001	1.20	1.05-1.38	0.01			
Encephalopathy	1.22	1.04-1.44	0.02	1.24	1.15-1.34	<0.001	1.19	1.01-1.40	0.04	1.14	1.06-1.23	0.001
Ascites	0.95	0.77-1.16	0.59	1.19	1.08-1.32	<0.001						
SBP	1.11	0.89-1.39	0.35	0.93	0.84-1.03	0.18						
Dialysis	1.43	1.20-1.71	<0.001	1.44	1.32-1.56	<0.001	1.24	0.99-1.54	0.06	1.41	1.27-1.56	<0.001
Previous abdominal surgery	1.08	0.94-1.24	0.26	1.14	1.07-1.22	<0.001				1.15	1.08-1.23	<0.001
MELD[c]	1.01	1.00-1.02	0.01	1.01	1.00-1.01	<0.001	1.01	0.99-1.02	0.37	1.02	1.01-1.02	<0.001
Serum albumin	1.00	0.91-1.10	0.99	0.90	0.86-0.94	<0.001				0.93	0.88-0.97	0.001
Serum bilirubin	1.01	1.00-1.02	0.001	1.01	1.00-1.01	<0.001	1.01	1.00-1.02	0.05			
INR	0.99	0.91-1.07	0.76	1.01	0.98-1.04	0.74						
Creatinine	1.13	1.08-1.19	<0.001	1.11	1.09-1.14	<0.001	1.07	1.01-1.14	0.03			
Sodium	1.03	1.01-1.04	<0.001	1.02	1.02-1.03	<0.001	1.02	1.00-1.03	0.01	1.02	1.01-1.02	<0.001
History of PVT (ref. PVT+)												
History of PVT-	1.22	0.99-1.52	0.07	1.12	0.97-1.30	0.13						
Unknown	1.17	0.65-2.13	0.60	1.48	1.24-1.77	<0.001						
Transplant year, (ref. 2002-2006)												

2007-2011	0.68	0.55-0.84	<0.001	0.79	0.72-0.85	<0.001	0.64	0.51-0.80	<0.001	0.71	0.65-0.77	<0.001
2012-2016	0.57	0.47-0.70	<0.001	0.54	0.49-0.60	<0.001	0.53	0.43-0.66	<0.001	0.47	0.42-0.51	<0.001
2017-2022	0.44	0.37-0.53	<0.001	0.42	0.38-0.45	<0.001	0.43	0.35-0.53	<0.001	0.41	0.37-0.44	<0.001
Geographic region, (ref. Northeast)												
Southeast	0.71	0.59-0.86	<0.001	0.75	0.69-0.81	<0.001	0.87	0.72-1.05	0.16	0.86	0.79-0.94	<0.001
Midwest	0.89	0.73-1.09	0.25	0.74	0.68-0.81	<0.001	1.02	0.84-1.25	0.82	0.84	0.76-0.92	<0.001
West	0.80	0.63-1.01	0.06	0.74	0.67-0.82	<0.001	0.86	0.67-1.09	0.21	0.77	0.70-0.86	<0.001
**Donor characteristics**												
Age[¶]	1.09	1.04-1.14	<0.001	1.12	1.10-1.14	<0.001	1.05	0.98-1.12	0.20	1.07	1.04-1.11	<0.001
Sex, female	1.22	1.07-1.39	0.004	1.10	1.03-1.17	0.01						
Race/ethnicity (ref. NH White)												
NH Black	0.97	0.81-1.15	0.70	1.10	1.01-1.20	0.03						
Hispanic	1.21	1.00-1.47	0.05	1.08	0.98-1.18	0.10						
NH Asian	1.05	0.67-1.65	0.82	1.17	0.95-1.44	0.15						
Other	0.70	0.31-1.57	0.39	0.90	0.64-1.27	0.56						
BMI	1.00	0.99-1.01	0.59	1.00	0.99-1.00	0.24						
BMI ≥ 30	0.96	0.84-1.11	0.61	0.92	0.85-0.98	0.02				0.92	0.86-1.00	0.04
Diabetes	1.37	1.15-1.64	<001	1.23	1.12-1.35	<0.001	1.30	1.08-1.57	0.01	1.14	1.04-1.26	0.01

CIT	1.08	1.05-1.11	<001	1.08	1.07-1.09	<0.001	1.04	1.02-1.07	0.002	0.99	0.98-0.99	<0.001
DRI				1.00	1.00-1.01	<0.001						
DRI categories (ref. Low-DRI)												
Medium-DRI	1.47	1.24-1.74	<001	1.34	1.24-1.46	<0.001	1.32	1.09-1.60	0.004	1.20	1.09-1.32	<0.001
High-DRI	1.37	1.18-1.60	<001	1.53	1.42-1.64	<0.001	1.14	0.88-1.47	0.33	1.22	1.08-1.38	0.001
DCD (ref. DBD)	0.98	0.76-1.27	0.90	0.75	0.66-0.87	<0.001	1.02	0.79-1.33	0.84	0.78	0.68-0.90	0.001
Donor-recipient match per BSA (ref. too small)												
Appropriate size	0.69	0.51-0.94	0.02	0.63	0.52-0.76	<0.001	0.63	0.46-0.87	0.004	0.65	0.54-0.79	<0.001
Too large	0.80	0.53-1.23	0.32	0.70	0.56-0.87	0.002	0.77	0.49-1.19	0.24	0.79	0.62-1.00	0.05

[a] Recipient and donor age divided by ten-years period.
[b] We stratified by diabetes and obesity on multivariate analysis to avoid violation of the proportionality hazard assumption.
[c] At the time of transplant.

Abbreviations: DBD, donation after brain death; DCD, donation after circulatory death; MASLD, metabolic dysfunction-associated steatotic liver disease; PVT, portal vein thrombosis; SD, standard deviation; NH, non-Hispanic; BMI, body mass index; SBP, spontaneous bacterial peritonitis; MELD, Model for End-Stage Liver Disease; IQR (inter-quartile range); INR, international normalized ratio; DRI, donor risk index; BSA, body surface area; CIT, cold ischemia time.

TABLE S4. Univariate and multivariate Cox regression analysis of death-censored graft failure in deceased-donor liver transplant MASLD and non-MASLD recipients between 2002 to 2022 in the U.S.

	Death-censored graft failure											
	UNIVARIATE						MULTIVARIATE					
	MASLD			Non-MASLD			MASLD			Non-MASLD[b]		
	aHR	95% CI	p-value	aHR	95% CI	p-value	aHR	95% CI	p-value	aHR	95% CI	p-value
**Recipient characteristics**												
PVT + (ref. PVT-)	1.34	1.01-1.78	0.040	1.19	1.03-1.39	0.022	1.50	1.12-2.01	0.007	1.38	1.20-1.59	<0.001
Age[a]	0.94	0.83-1.07	0.345	1.01	0.97-1.06	0.616	1.04	0.91-1.20	0.569	1.01	0.96-1.05	0.813
Female sex	0.82	0.66-1.03	0.092	0.94	0.85-1.03	0.173	0.88	0.69-1.11	0.270	0.86	0.79-0.95	0.002
Race (ref. NH White)												
Black	1.10	0.54-2.22	0.798	1.41	1.22-1.62	<0.001				1.39	1.21-1.61	<0.001
Hispanic	0.75	0.53-1.07	0.115	0.87	0.75-1.00	0.054				0.88	0.77-1.01	0.067
Asian	0.88	0.36-2.13	0.779	0.81	0.61-1.09	0.166				0.79	0.60-1.05	0.104
Other	0.80	0.30-2.14	0.655	0.97	0.66-1.45	0.898				1.19	0.82-1.72	0.357
Blood type (ref. O)												
A	0.97	0.75-1.24	0.790	0.96	0.87-1.07	0.483						
B	1.23	0.89-1.71	0.212	1.04	0.91-1.20	0.554						
AB	1.03	0.62-1.71	0.899	1.03	0.84-1.27	0.773						

BMI	1.03	1.01-1.05	0.002	1.01	0.99-1.01	0.108	1.02	1.00-1.04	0.124	0.99	0.97-1.02	0.881
BMI ≥ 30	1.72	1.29-2.30	<0.001	1.05	0.95-1.16	0.312				0.83	0.74-0.92	<0.001
Diabetes	1.12	0.90-1.40	0.312	1.27	1.12-1.43	<0.001	1.21	0.96-1.52	0.105			
Encephalopathy	1.25	0.95-1.65	0.111	1.17	1.05-1.30	0.004	1.26	0.95-1.67	0.103	1.17	1.06-1.29	0.003
Ascites	0.96	0.68-1.35	0.809	1.19	1.03-1.37	0.019						
SBP	0.87	0.59-1.31	0.513	0.73	0.62-0.86	<0.001						
Dialysis	0.93	0.65-1.32	0.690	0.98	0.85-1.13	0.782	1.01	0.65-1.55	0.981	1.25	1.07-1.46	0.005
Previous abdominal surgery	0.90	0.72-1.13	0.365	1.16	1.06-1.27	0.002				1.2	1.10-1.31	<0.001
MELD§	0.99	0.98-1.00	0.113	0.99	0.98-0.99	<0.001	1.00	0.98-1.02	0.982	1.01	1.00-1.01	0.004
Serum albumin	1.02	0.87-1.21	0.769	0.77	0.72-0.82	<0.001				0.92	0.87-0.98	0.015
Serum bilirubin	1.01	1.00-1.02	0.153	0.90	0.99-1.00	0.154	1.01	1.00-1.03	0.071			
INR	0.95	0.83-1.09	0.487	0.91	0.87-0.96	0.001						
Creatinine	1.01	0.92-1.12	0.783	1.05	1.02-1.09	0.006	0.99	0.87-1.12	0.841			
Sodium	1.02	1.00-1.05	0.035	1.03	1.02-1.03	<0.001	1.02	0.99-1.04	0.208	1.02	1.01-1.03	0.001
History of PVT (ref. PVT+)												
History of PVT-	1.02	0.70-1.50	0.906	0.83	0.65-1.05	0.126						
Unknown	2.01	0.83-4.87	0.121	2.12	1.68-2.69	<0.001						

Transplant year, (ref. 2002-2006)												
2007-2011	0.44	0.32-0.61	<0.001	0.55	0.49-0.62	<0.001	0.44	0.32-0.62	<0.001	0.52	0.47-0.58	<0.001
2012-2016	0.23	0.17-0.32	<0.001	0.29	0.26-0.34	<0.001	0.26	0.18-0.36	<0.001	0.27	0.24-0.32	<0.001
2017-2022	0.15	0.11-0.20	<0.001	0.19	0.16-0.21	<0.001	0.17	0.13-0.24	<0.001	0.2	0.17-0.22	<0.001
Geographic region, (ref. Northeast)												
Southeast	0.62	0.45-0.86	0.004	0.68	0.61-0.77	<0.001	0.81	0.58-1.12	0.202	0.78	0.70-0.88	<0.001
Midwest	0.98	0.71-1.36	0.904	0.75	0.65-0.85	<0.001	1.16	0.83-1.63	0.369	0.91	0.81-1.03	0.15
West	0.65	0.43-0.98	0.039	0.67	0.57-0.77	<0.001	0.82	0.54-1.26	0.372	0.77	0.67-0.89	<0.001
**Donor characteristics**												
Age[c]	1.16	1.08-1.25	<0.001	1.22	1.18-1.25	<0.001	1.04	0.92-1.17	0.553	1.11	1.06-1.16	<0.001
Sex, female	1.30	1.04-1.63	0.020	1.19	1.09-1.31	<0.001						
Race/ethnicity (ref. NH White)												
NH Black	1.15	0.87-1.52	0.342	1.14	1.01-1.28	0.032						
Hispanic	1.06	0.75-1.50	0.730	1.04	0.90-1.19	0.608						
NH Asian	1.17	0.58-2.36	0.670	1.11	0.81-1.52	0.525						
Other	0.67	0.17-2.68	0.568	0.78	0.46-1.32	0.349						
Donor BMI	0.99	0.97-1.00	0.151	0.99	0.98-1.00	0.056						

Donor BMI ≥ 30	0.82	0.65-1.05	0.119	0.83	0.75-0.92	0.001						
Diabetes	1.62	1.23-2.15	0.001	1.43	1.26-1.63	<0.001	1.48	1.10-1.99	0.009	1.3	1.15-1.47	<0.001
CIT	1.15	1.11-1.19	<0.001	1.14	1.13-1.16	<0.001	1.08	1.04-1.13	<0.001	0.97	0.96-0.98	<0.001
DRI	1.38	1.20-1.58	<0.001	1.51	1.42-1.60	<0.001						
DRI categories (ref. Low-DRI)												
Medium-DRI	1.86	1.41-2.47	<0.001	1.68	1.50-1.89	<0.001	1.68	1.22-2.32	0.002	1.5	1.33-1.71	<0.001
High-DRI	1.78	1.37-2.31	<0.001	2.05	1.84-2.27	<0.001	1.40	0.91-2.16	0.123	1.45	1.23-1.71	<0.001
DCD (ref. DBD)	0.99	0.64-1.52	0.97	0.77	0.63-0.95	0.013	1.05	0.68-1.63	0.81	0.84	0.69-1.03	0.10
Donor-recipient match per BSA (ref. too small)												
Appropriate size	0.41	0.27-0.61	<0.001	0.53	0.41-0.68	<0.001	0.45	0.29-0.68	<0.001	0.54	0.44-0.68	<0.001
Too large	0.35	0.17-0.70	0.003	0.51	0.37-0.69	<0.001	0.52	0.25-1.09	0.082	0.68	0.51-0.92	0.012

[a] Recipient and donor age divided by ten-years period.
[b] We stratified by diabetes and obesity on multivariate analysis to avoid violation of the proportionality hazard assumption.
[c] At the time of transplant.

Abbreviations: DBD, donation after brain death; DCD, donation after circulatory death; MASLD, metabolic dysfunction-associated steatotic liver disease; PVT, portal vein thrombosis; SD, standard deviation; NH, non-Hispanic; BMI, body mass index; SBP, spontaneous bacterial peritonitis; MELD, Model for End-Stage Liver Disease; IQR (inter-quartile range); INR, international normalized ratio; DRI, donor risk index; BSA, body surface area; CIT, cold ischemia time.

Table S5. Causes of death within the first year after liver transplantations in patients with MASLD and Non-MASLD, stratified by PVT status

		MASLD		Non-MASLD		
**Complication**	**Total**	**PVT+**	**PVT-**	**PVT+**	**PVT-**	**p-value**
						< 0.01
**Cardiovascular**	653 (21.0)	36 (29.0)	109 (23.0)	63 (21.6)	445 (20.1)	
**Infection**	731 (23.5)	27 (21.8)	122 (25.7)	72 (24.7)	510 (23.0)	
**MOSF**	421(13.6)	14 (11.3)	68 (14.3)	36 (12.3)	303 (13.7)	
**Malignancy**	85 (2.7)	2 (1.6)	12 (2.5)	8 (2.7)	63 (2.9)	
**Respiratory**	192 (6.2)	13 (10.5)	41 (8.6)	16 (5.5)	122 (5.5)	
**Graft failure**	406 (13.1)	11 (8.9)	38 (8.0)	43 (14.7)	314 (14.2)	
**Renal failure**	35 (1.1)	0 (0)	8 (1.7)	4 (1.4)	23 (1.0)	
**Other**	372 (12.0)	16 (12.9)	51 (10.7)	33 (11.3)	272 (12.3)	
**Unknown**	210 (6.8)	5 (4.0)	26 (5.5)	17 (5.8)	162 (7.3)	

Abbreviations: MASLD, metabolic dysfunction-associated liver disease; PVT, portal vein thrombosis; MOSF, multi-organ system failure. Other included the following complications: operative, trauma, diabetes, acid/base disorder, non-immune drug related and other specific.

Table S6. Causes of graft failure in recipients with MASLD and Non-MASLD undergoing transplantation, stratified by PVT status

**Etiologies**		**MASLD**		**Non-MASLD**		
**PVT Status**	**Total**	**PVT+**	**PVT-**	**PVT+**	**PVT-**	**p-value**
						< 0.01
**Primary non function**	505 (37.4)	18 (41.9)	76 (42.2)	49 (40.5)	362 (36.0)	
**Biliary complications**	152 (11.3)	3 (7.0)	23 (12.8)	14 (11.6)	112 (11.1)	
**Recurrence of disease**	80 (5.9)	0 (0)	4 (2.2)	5 (4.1)	71 (7.1)	
**Acute rejection**	97 (7.2)	1 (2.3)	10 (5.6)	8 (6.6)	78 (7.8)	
**Chronic rejection**	95 (7.0)	3 (7.0)	9 (5.0)	6 (5.0)	77 (7.7)	
**Infection**	289 (21.4)	11 (25.6)	43 (23.9)	24 (19.8)	211 (21.0)	
**Hepatic artery thrombosis**	132 (9.8)	7 (16.3)	15 (8.3)	15 (12.4)	95 (9.4)	

Abbreviations: MASLD, metabolic dysfunction-associated liver disease; PVT, portal vein thrombosis.

Figure S1. Prevalence of PVT in LT recipients across 5-year intervals periods. (A) MASLD and non-MASLD. (B) MASLD and non-MASLD stratified by etiologies. * for significant p-value. Abbreviations: AILDs, autoimmune liver disease; ALD, alcoholic liver disease; HBV, hepatitis B; HCV, hepatitis C; LT, liver transplantation; MASLD, metabolic dysfunction-associated steatotic liver disease; PVT, portal vein thrombosis.

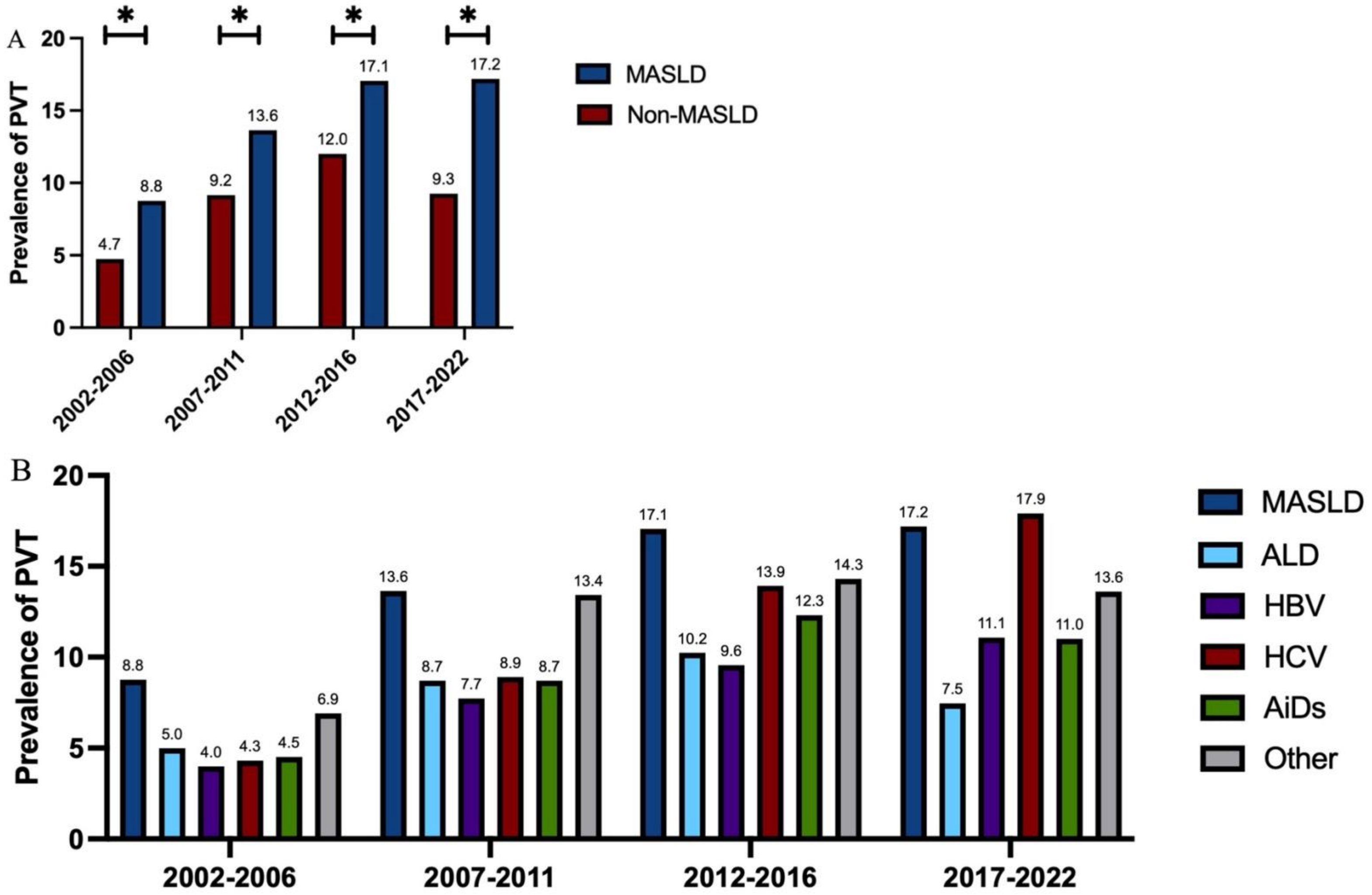

Figure S2. Kaplan–Meier curves of patient survival after one-year post-LT in patients with MASLD and Non-MASLD, stratified by presence or absence of PVT across 5-year interval periods: (A) 2002-2006 (B) 2007-2011 (C) 2012-2016 (D) 2017-2022. Abbreviations: LT, liver transplantation; MASLD, metabolic dysfunction-associated steatotic liver disease; PVT, portal vein thrombosis.

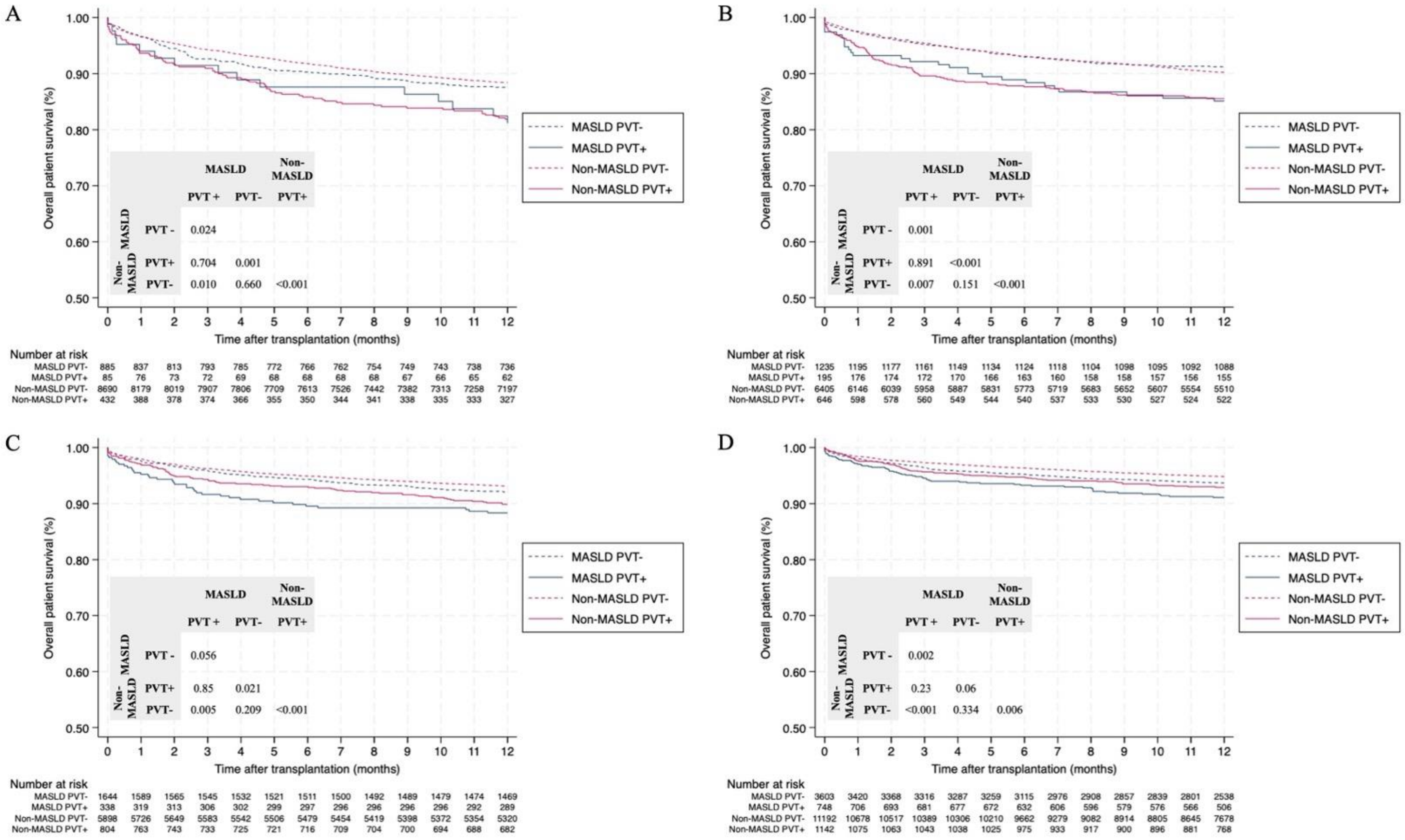

Figure S3. Kaplan–Meier curves of graft survival after one-year post-LT in patients with MASLD and Non-MASLD, stratified by presence or absence of PVT across 5-year interval periods: (A) 2002-2006 (B) 2007-2011 (C) 2012-2016 (D) 2017-2022. Abbreviations: LT, liver transplantation; MASLD, metabolic dysfunction-associated steatotic liver disease; PVT, portal vein thrombosis.

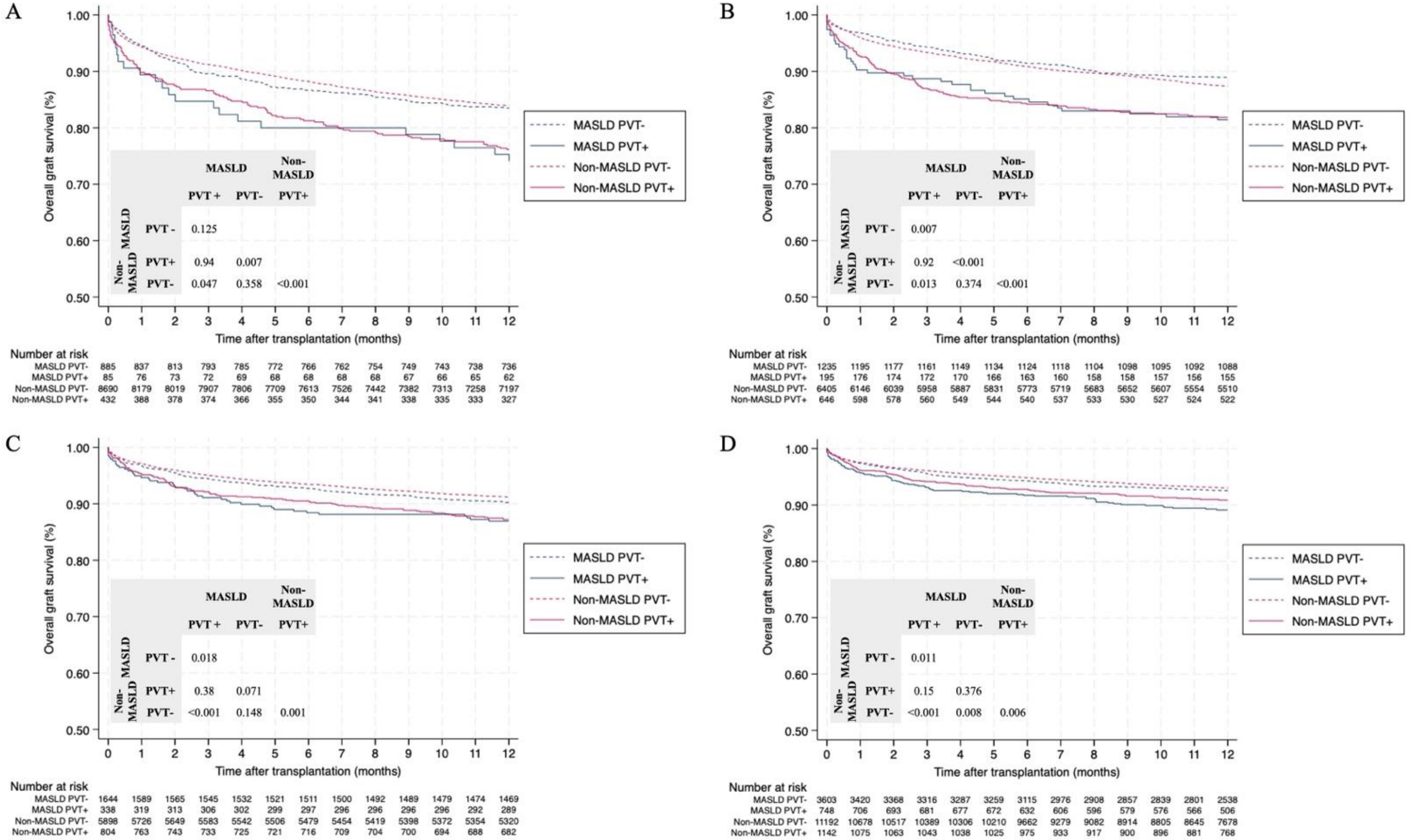

Figure S4. Kaplan–Meier curves of death-censored graft survival after one-year post-LT in patients with MASLD and Non-MASLD, stratified by presence or absence of PVT across 5-year interval periods: (A) 2002-2006 (B) 2007-2011 (C) 2012-2016 (D) 2017-2022. Abbreviations: LT, liver transplantation; MASLD, metabolic dysfunction-associated steatotic liver disease; PVT, portal vein thrombosis.

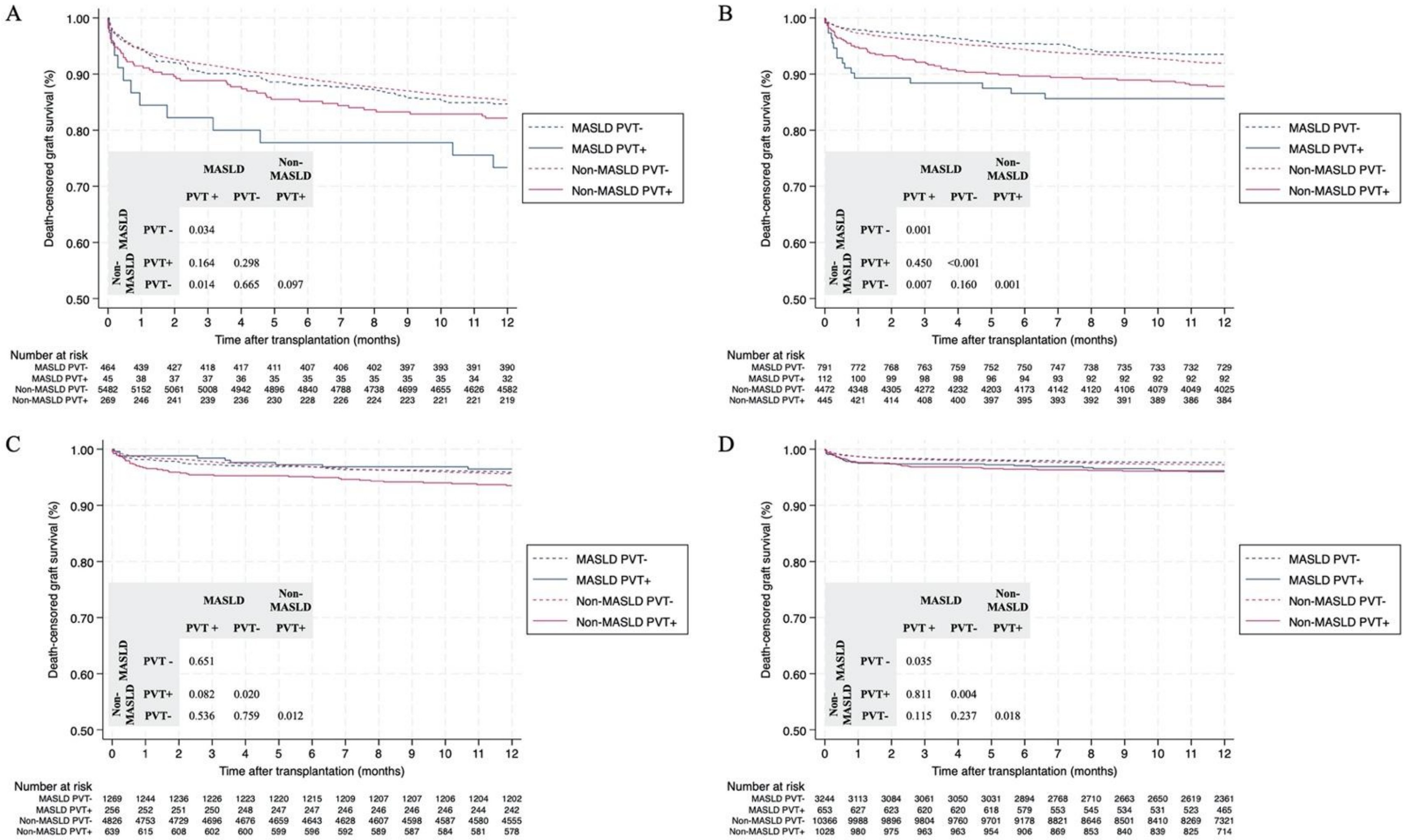

Figure S5. Kaplan–Meier curves of (A) overall patient and (B) graft survival and (C) death-censored graft survival after LT in patients with PVT at time of transplant, stratified by etiology of liver disease. Abbreviations: AILDs, autoimmune liver disease; ALD, alcoholic liver disease; HBV, hepatitis B; HCV, hepatitis C; LT, liver transplantation; MASLD, metabolic dysfunction-associated steatotic liver disease; PVT, portal vein thrombosis.

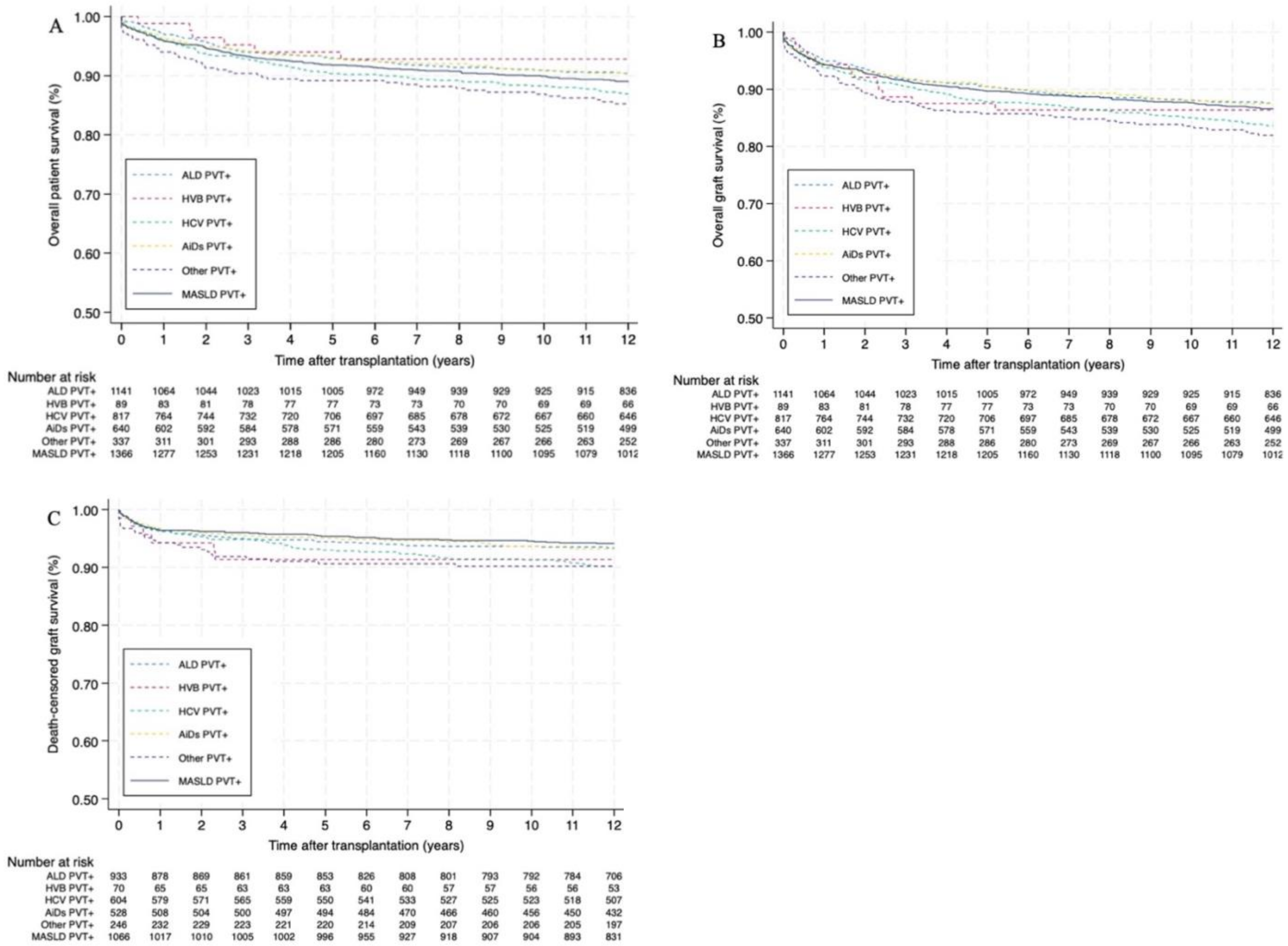

Figure S6. Kaplan–Meier curves of overall patient survival (A, B), graft survival (C, D) and death-censored graft survival (E, F) after one-year post-LT in patients with MASLD and non-MASLD with and without PVT at listing (PVT Hx) and at transplant (PVT Tx).

Abbreviations: LT, liver transplantation; MASLD, metabolic dysfunction-associated steatotic liver disease; PVT, portal vein thrombosis.

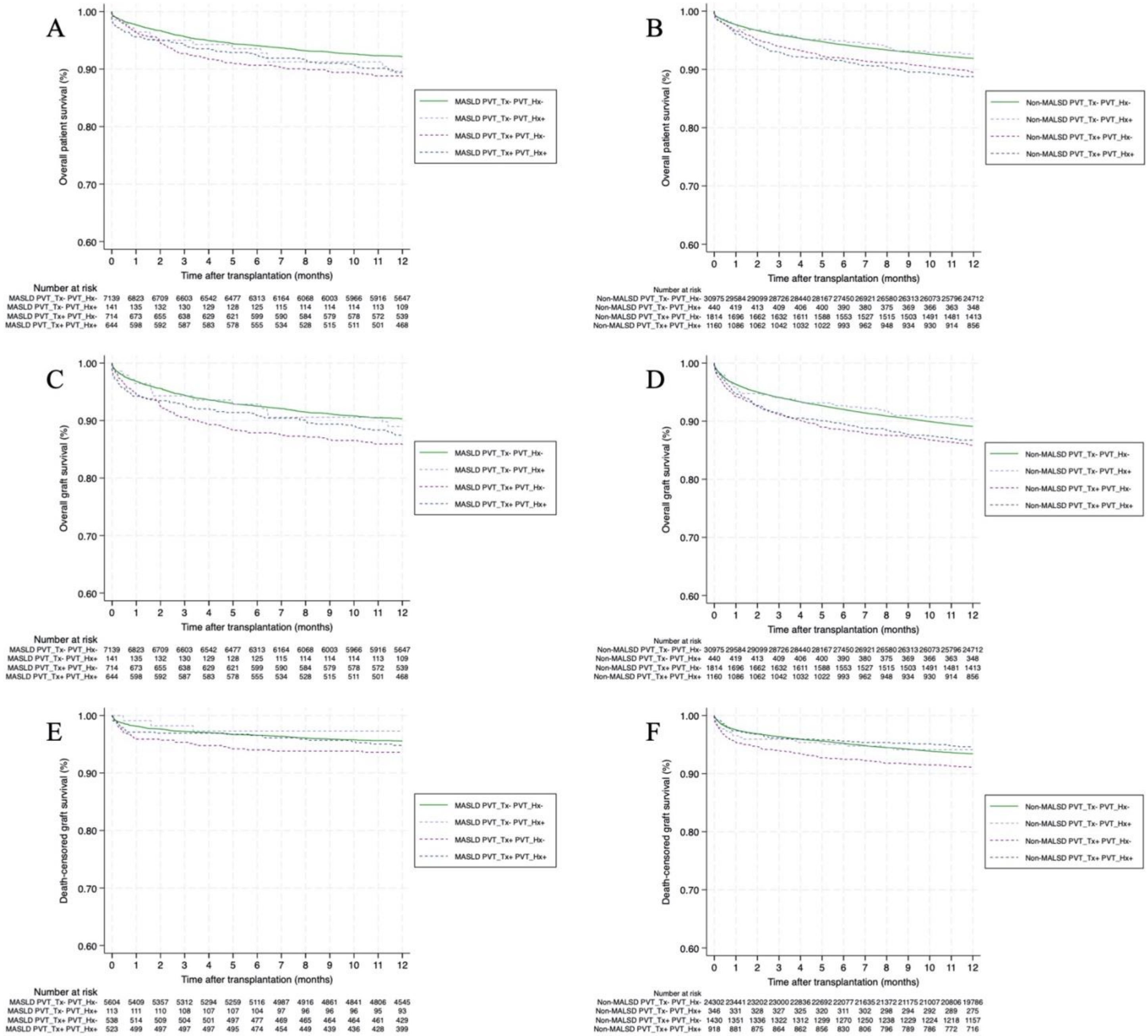

Supplement: Supplementary file 1 [file jcm-15-01787-s001.zip › jcm-4143558-supplementary.pdf]
